# *PLATINUM SENSITIVE 2 LIKE* impacts growth, root morphology, seed set, and stress responses

**DOI:** 10.1371/journal.pone.0180478

**Published:** 2017-07-05

**Authors:** Amr R. A. Kataya, Maria T. Creighton, Toga P. Napitupulu, Christine Sætre, Behzad Heidari, Peter Ruoff, Cathrine Lillo

**Affiliations:** Centre for Organelle Research, Faculty of Science and Technology, University of Stavanger, Stavanger, Norway; National Taiwan University, TAIWAN

## Abstract

Eukaryotic protein phosphatase 4 (PP4) is a PP2A-type protein phosphatase that is part of a conserved complex with regulatory factors PSY2 and PP4R2. Various lines of *Arabidopsis thaliana* with mutated *PP4* subunit genes were constructed to study the so far completely unknown functions of PP4 in plants. Mutants with knocked out putative functional homolog of the *PSY2 LIKE (PSY2L)* gene were dwarf and bushy, while plants with knocked out *PP4R2 LIKE (PP4R2L)* looked very similar to WT. The *psy2l* seedlings had short roots with disorganized morphology and impaired meristem. Seedling growth was sensitive to the genotoxin cisplatin. Global transcript analysis (RNA-seq) of seedlings and rosette leaves revealed several groups of genes, shared between both types of tissues, strongly influenced by knocked out *PSY2L*. Receptor kinases, *CRINKLY3* and *WAG1*, important for growth and development, were down-regulated 3–7 times. *EUKARYOTIC ELONGATION FACTOR5A1* was down-regulated 4–6 fold. Analysis of hormone sensitive genes indicated that abscisic acid levels were high, while auxin, cytokinin and gibberellic acid levels were low in *psy2l*. Expression of specific transcription factors involved in regulation of anthocyanin synthesis were strongly elevated, e.g. the master regulator *PAP1*, and intriguingly *TT8*, which is otherwise mainly expressed in seeds. The *psy2l* mutants accumulated anthocyanins under conditions where WT did not, pointing to PSY2L as a possible upstream negative regulator of PAP1 and TT8. Expression of the sugar-phosphate transporter *GPT2*, important for cellular sugar and phosphate homeostasis, was enhanced 7–8 times. Several DNA damage response genes, including the cell cycle inhibitor gene *WEE1*, were up-regulated in *psy2l*. The activation of DNA repair signaling genes, in combination with phenotypic traits showing aberrant root meristem and sensitivity to the genotoxic cisplatin, substantiate the involvement of Arabidopsis PSY2L in maintenance of genome integrity.

## Introduction

Protein phosphatase 4 (PP4) is a highly conserved serine/threonine protein phosphatase in eukaryotes. PP4 belongs to the PP2A type of protein phosphatases, and like PP2A, appears in complexes with specific regulatory and scaffolding subunits. PP2A is present in the cell as a dimer and trimer. The canonical dimer is made up of a catalytic and a scaffolding subunit. A third regulatory subunit is joined to form the active holoenzyme. Complexes with catalytic, scaffolding and regulatory subunits are found also for PP4 [1, 2]. However, for PP4, a scaffolding subunit is not always present. A conserved PP4 complex is found in all eukaryotes, from yeast, to mammals and plants. This conserved PP4 heterotrimer consists of a catalytic and two regulatory/scaffolding subunits. In *Saccharomyces cerevisiae* these are called Pph3, Psy2 and Ybl046, and in mammals PP4c, PP4R3 and PP4R2. Some studies performed with yeast and mammals have shown that the PP4 catalytic subunit and Psy2/PP4R3, but not Yb1046/PP4R2, can be crucial for regulation of specific biological processes [3–5]. Apparently both trimeric and dimeric active forms of PP4 exist in vivo. In mammals, two other regulatory subunits, PP4R1 and PP4R4, were also identified, but orthologues of these have not been found in yeast or plants [1, 6–8].

Arabidopsis has two genes encoding the PP4 catalytic (PP4c) subunit, PP4-1 (At4g26720) and PP4-2 (At5g55260), which are 94% identical at the amino acid level. With some variations, both genes are expressed throughout all plant organs. Arabidopsis has one gene, *PP4R2L* (*PP4R2 LIKE*) (At5g17070) encoding a PP4R2 domain protein, also expressed throughout the plant (TAIR database, eFP Browser; [9]). *PP4R2L* functions likely as the mammalian PP4R2, in a trimeric PP4 complex. Arabidopsis PP4R2 has 32% identity (coverage 48%) with the human protein. The Arabidopsis protein is smaller, consisting of 277 amino acids (30 kD), whereas the human protein has 417 amino acids (47 kD) [8].

PSY2 (also called PP4R3, Falafel, and SMK1) is conserved in eukaryotes. Arabidopsis PSY2L (PLATINUM SENSITIVE 2 LIKE) (At3g06670) protein consists of 865 amino acids and has a molecular mass of 97 kD. The coding sequence is made up of 24 exons (RNA-seq did not give evidence for a splice 2 variant). PSY2L is a highly conserved protein with 35% identity (coverage 78%) with human PP4R3, and has several domains conserved among eukaryotes. The PH (Pleckstrin homology)/EVH1 (Enabled/vasodilator-stimulated phosphoprotein homology 1) domain is located in the N-terminal end, amino acid 15–113 in Arabidopsis PSY2L. Strikingly, this domain is 56% identical and 70% similar in Arabidopsis and mammals. PH-domain proteins were originally associated with phospholipids and membrane interactions, but recent examples showing the importance in protein-protein interactions have by far exceeded the phospholipid interactions [10]. In the fruit fly *Drosophila melanogaster*, the PH/EVH1 domain of PSY2 (Falafel) was found to bind to the centromeric protein C (CENP-C), and was important to bridge the centromere to kinetochore proteins to sustain proper chromosome segregation during the cell cycle [11]. The other highly conserved domain in PSY2L is domain of unknown function DUF625 (also called SMK1) positioned at amino acids 166–356 in Arabidopsis PSY2L. The Arabidopsis SMK1 domain is 42% identical (66% similar) to the human PP4R3 SMK1 domain. The SMK1 domain is named after SMEK (suppressor of MEK null), initially identified in the slime mold *Dictyostelium discoideum* [12] and the worm *Caenorhabditis elegans* [13]. *C*. *elegans* SMK1 protein was found to be part of the IIS longevity pathway, which regulates larval arrest and aging [13]. The N-terminal domains are followed by a conserved Armadillo-type fold, approximately covering 300 amino acids, and functional by assembling into superhelical structures suitable for binding other proteins [14, 15]. Arabidopsis *PSY2L* gene is expressed in vegetative and reproductive organs at a relatively high level throughout the life cycle of the plant (TAIR database, eFP Browser; [9]). Hence all putative Arabidopsis *PP4* subunits, the two catalytic and the two regulatory, are expressed throughout the plant.

In *S*. *cerevisiae* it was shown that the dimer PP4c-PSY2 (named Pph3-Psy2 in yeast) is involved in regulating HXT genes (glucose transporter genes). For this regulation the PH/EVH1 domain of PSY2 is important and was found to interact with glucose signaling transducer protein (Mth1) [16]. In mammals, PSY2 is engaged in the regulation of glucose metabolism, and in the regulation of phosphorylation state of a transcription activator CRTC2 (CREB-regulated transcriptional coactivator 2) [17]. In *C*. *elegans* the PSY2 was also involved in control of sugar metabolism because the IIS longevity pathway is activated through the insulin/IGF-1 receptor (DAF-2). PSY2 is part of this pathway by regulating FOXO transcription factor (DAF-16) downstream in the pathway [13]. In the present work we show importance of *PSY2L* for expression of a key sugar transporter gene in Arabidopsis.

Cisplatin is a platinum-containing DNA damaging agent and a drug used to treat cancer. PSY2 was originally identified in yeast cells when selecting drug-sensitive strains [18], and named Platinum sensitive 2. Drosophila mutated in the homologous gene (falafel) also showed cisplatin sensitivity, e.g. had reduced survival rate when exposed to cisplatin [2]. As for other eukaryotes, also in plants, cisplatin sensitivity has been shown to involve defects in DNA repair. At the plant organ level, exposure to cisplatin inhibits leaf formation and growth [19, 20].

Nothing is known about the physiological function of PP4c and its two putative regulatory proteins PSY2L and PP4R2L in plants. We embarked to investigate the functions of these genes by selecting T-DNA insertion mutants and by making RNA interference lines. Interestingly, mutants of Arabidopsis *PSY2L* showed a striking visual phenotype and sensitivity to cisplatin. Additionally, putative genes and pathways regulated by PSY2L were revealed by RNA sequencing.

## Results

### Phenotype of PP4 subunit mutants—Impaired *PSY2L* leads to slow growth, dwarfism, sterility and longevity

**Fig 1:**
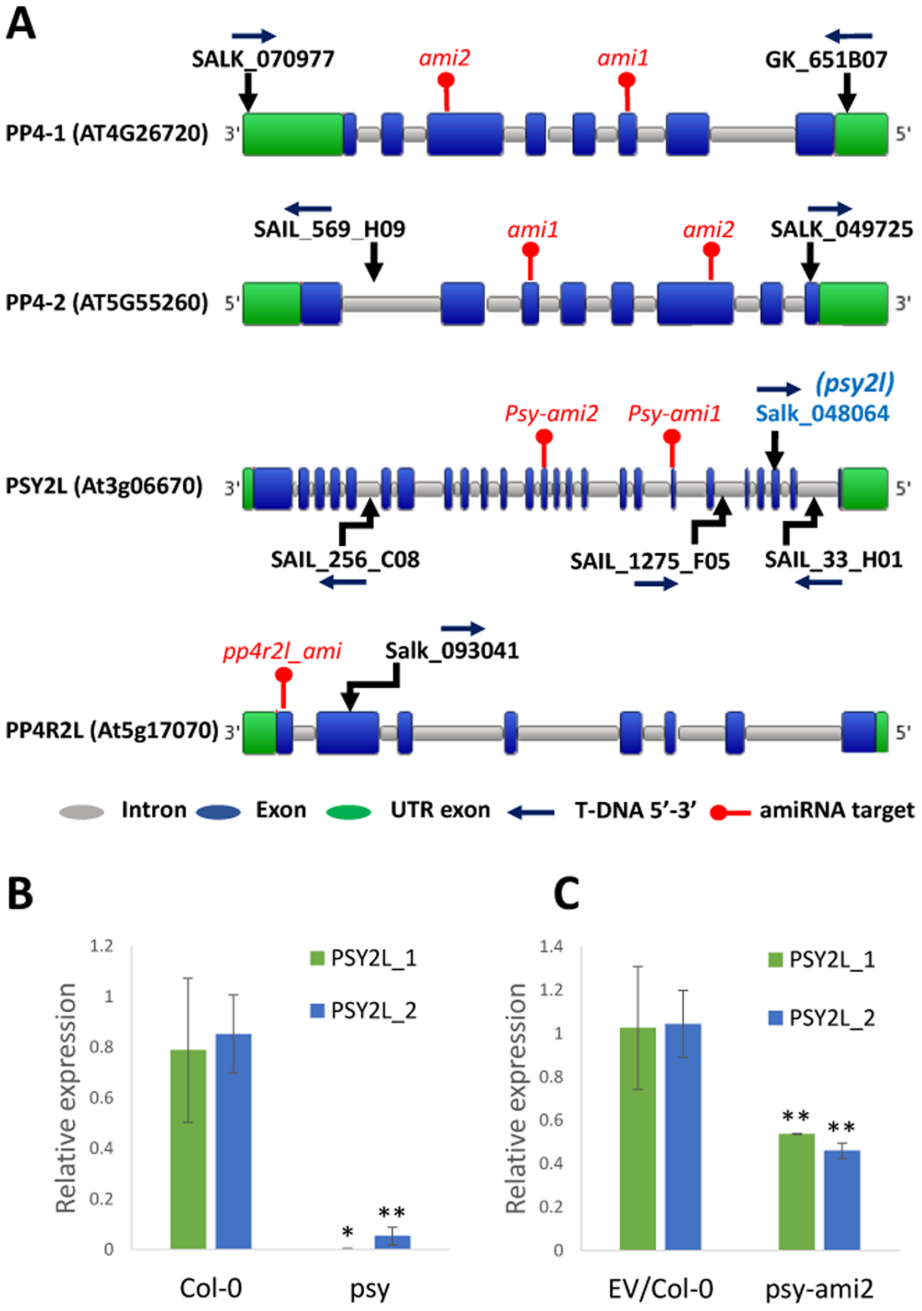
Schemes for target sites of T-DNA and amiRNA, and expression analysis of *PSY2L* in the SALK_049725 line and psy-ami2 line. **A**, T-DNA insertion lines. The target sites and orientation of T-DNA insertions are indicated. The insertion line Salk_048064 (*psy2l*) was used in most studies. Target sites of amiRNAs are indicated with a red mark. ami1 targeted exon 3 in both *PP4-1* and *PP4-2* genes, and ami2 targeted exon 6 in both genes. Schemes are from the PLAZA database [21]. **B**, Quantitative real time expression analysis of the *PSY2L* gene in WT (Col-0) and the SALK_048064 line tested with two different primer pairs spanning exons 18–19 (green columns) or exons 3 and 4 (blue columns). **C**, Quantitative real time expression analysis of the *PSY2L* gene in EV/Col-0 (plants transformed with empty vector) and the psy-ami2 line. RNA from three replicates of soil-grown plants (four weeks old) was used. SE is given, Expression in mutant lines is significant different from (EV)/Col0 at the level: * p<0.05, ** p<0.01.

In order to investigate PP4 functions, two homozygous T-DNA insertion lines were isolated for both *PP4-1* and *PP4-2* (Fig 1A). However, PP4c transcript levels in all four lines were similar to WT transcript levels. Two amiRNA lines for simultaneous knockdown/out of both *PP4-1* and *PP4-2* genes were made (Fig 1A). The lines, with constitutive (35S driven) expression of microRNAs, were followed until the fourth generation. Extensive expression analysis gave five positive knockdown plants, however, their progeny reverted to normal WT expression levels, indicating difficulties with isolation of stable knockdown/out lines for the catalytic subunits (data not presented). No clear phenotype was observed in any of the generations, and sufficient PP4c was apparently present to support normal growth and development.

**Fig 2:**
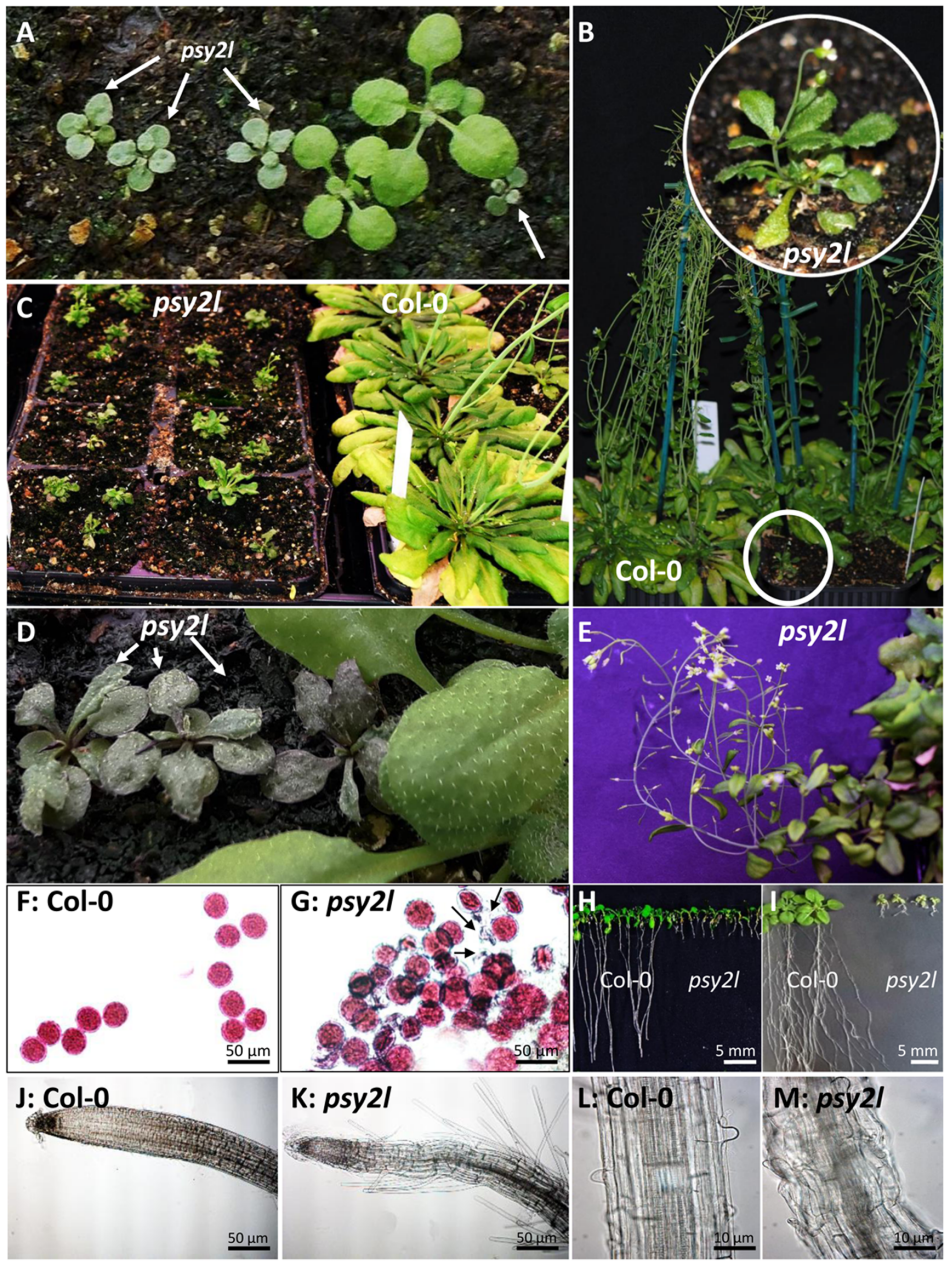
Phenotype of the *psy2l* knockout line (SALK_048064). **A**, Three weeks old homozygous *psy2l* (arrows), verified by genotyping, had a distinct appearance as compared with WT (the two bigger plants). **B-C**, Ten weeks old *psy2l* showed dwarfism and late flowering. **D**, Anthocyanin accumulation in four weeks old *psy2l* while WT leaves to the right do not accumulate anthocyanins. **E**, Eight months old semi-sterile *psy2l*. Most of the siliques contained either few or no seeds. **F-G**, Alexander staining of viable pollen. In wild type, clearly all pollen was stained. In *psy2l* most of the pollen was stained, also some non-stained pollen was seen. **H**, **I**, Eight and 20 days old seedlings showing severe retardation in root and shoot development in *psy2l* compared with WT. **J-M**, Images of roots from 10–12 days old WT and *psy2l* plants showing abnormal roots for *psy2l* with extensive root hairs close to the root tip and disorganized root cells. In A-G and H-M, plants were grown under 12 h light/ 12 h dark and 16 h light/ 8 h dark conditions, respectively.

Mutants homozygous for T-DNA insert in the *PSY2L* gene (SALK_040864) were isolated (*psy2l* line), and RT-PCR analysis confirmed complete knockout of *PSY2L* (Fig 1B). The *psy2l* plants were dwarf, and extremely slow growing (Fig 2A–2E). They grew into small bushy plants producing many flowering stems with poor silique development and very few seeds per plant. Three other homozygous mutant lines (SAIL_1275_F05, SAIL_33_H01, SAIL_256_C08, Fig 1A) showed the same dwarfed phenotype and development into small bushy plants with very poor seed set, hence confirmed that impairment of *PSY2L* causes such phenotype traits (S1 and S2 Figs).

**Fig 3:**
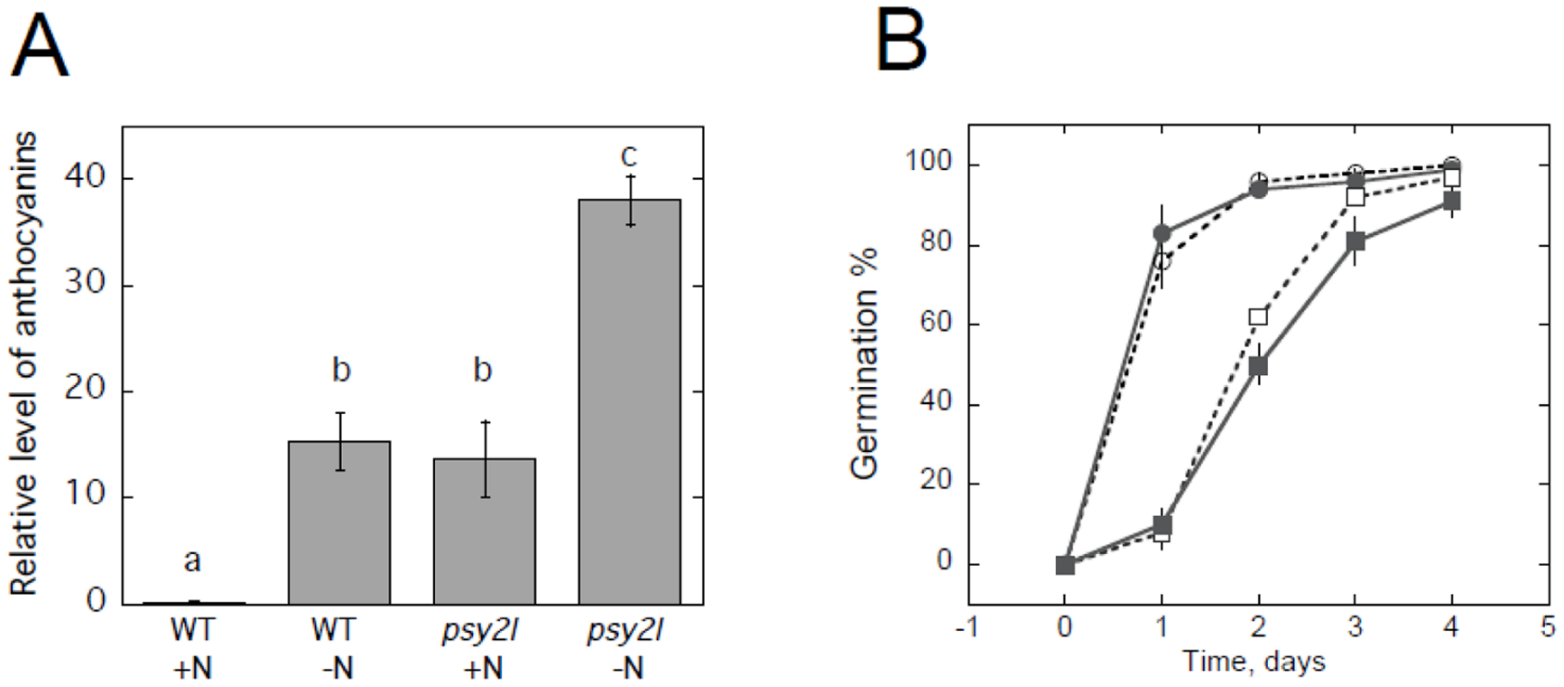
Anthocyanins in shoots, and germination time. **A**, Anthocyanin levels in shoots of WT and *psy2l* grown in rock wool with complete nutrient solution (+N) or solution without KNO_3_ (-N) for 1 week. Before this treatment plants had been grown for 5 weeks in rock wool with complete nutrient solution. n = 3, SE is given. Statistically significant differences (p<0.02) are indicated by different letters above the bars. **B**, Seed germination of WT (circles) and *psy2l* (squares) sown on ½ MS salts with 1% sucrose without (open symbols) or with 5 μM gibberellic acid (closed symbols). After sowing, seeds had been stratified at 5°C for three days, then placed at 22°C in 16 h light/8 h darkness. Totally there were 90 seeds for each treatment and plant type, e.g. three repeats each with 30 seedlings, n = 3, SE is given. On day 1 and 2 *psy2l* is significantly different from WT with p<0.01. GA effects were not significant.

The *psy2l* mutants easily developed purple colored leaves typical for high anthocyanin content (Fig 2D). WT plants growing on rock wool with complete nutrient medium did not have visible anthocyanins as confirmed by measurements (Fig 3A). Anthocyanins accumulated in WT grown on nitrogen-depleted nutrient solution, as expected [22]. However, for *psy2l*, the anthocyanin level was high already on the complete nutrient medium, and was then further enhanced by low nitrogen in the growth medium (Fig 3A). After seven months, the *psy2l* plants were still flowering, and showed complete or partial sterility (Fig 2E). Alexander staining [23] indicated viable cytoplasm and some aborted pollen in the *psy2l* plants (Fig 2G). Although most of the mutant pollen did stain red, differences from WT were obvious. Counting pollen grains in the microscope from ten intact anthers of WT and *psy2l* revealed a decrease in number by 58 ±6% in *psy2l*. Furthermore, from WT anthers pollen easily shed onto the microscope slide whereas mutant pollen did not. The oval shape of ripe pollen grains, was clearly seen for WT, but seldom found for the mutant pollen grains (S3 Fig), and there was much less pollen germinating from *psy2l*. Apparently much less pollen was able to interact properly with the stigma and lead to seed formation in *psy2l* as compared with WT. When *psy2l* seeds were sown on ½MS agar with 1% sucrose, impaired root growth was striking (Fig 2H), and also shoots were smaller (Fig 2I). *Psy2l* seedling roots clearly differed from WT by having root hairs closer to the tip of the root (Fig 2J and 2K) and disorganized structure (Fig 2L and 2M), resembling roots of mutants with impaired DNA double strand break repair [24]. When roots were stained with propidium iodide and examined by confocal microscopy it was clearly seen that the *psy2l* mutant had an aberrant meristem, e.g. shorter meristem zone with many dead cells (S4 Fig). In comparison with WT, *psy2l* showed significant delayed germination after 1 or 2 days at room temperature (Fig 3B), which could be caused by low concentration of GA or enhanced ABA levels. Delayed germination was highly reproducible with different batches of seeds. Germination was also tested in the presence of 5 μM gibberellic acid, but this did not significantly influence germination (Fig 3B). Other concentrations of gibberellic acid (1 and 10 μM) were also tested, but gave no positive effects (data not shown).

Because of the severe phenotypes of *psy2l* T-DNA knockout mutants, we also generated two microRNA (amiRNA) encoding genes that target exon 7 and 13 (Fig 1A). These lines showed knockdown by 50% of *PSY2L* transcripts (Fig 1C). Interestingly, plants from both ami-RNA1 and ami-RNA2 lines showed visible phenotypes, with different rosette appearance, shorter roots and flowering delay, but mild effects in comparison with the T-DNA knockout mutants of *PSY2L*. For example, mean root length of seedlings grown six days in vertical Petri dishes was 20.0 ± 0.7 mm for WT control (transformed with empty plasmid), 5.0 ± 0.4 mm for the *psy2l* SALK line, and 16.3 ± 0.4 mm and 15.6 ± 0.3 mm for *psy-ami1* and *psy-ami2*, respectively (S5 Fig).

Expression of *PP4R2L* in the homozygous mutant of *pp4r2l* (SALK_093041, Fig 1A) was tested with different primer pairs. The primer pair targeting upstream of the T-DNA insert showed over-expression, while the primer pair targeting downstream of the insert or spaning the full CDS showed knockdown of the transcript (Fig 1A, S6A Fig). The mutant showed no visual phenotype. An amiRNA complete knockout line for PP4R2L was generated (Fig 1A, S6B Fig), but also did not show any striking phenotype. Possibly, there was a mild accelerated senescence-like phenotype for cauline leaves that needs to be carefully investigated in the future. The severe phenotype of knockout *psy2l* mutants as opposed to the WT-like phenotype of *pp4r2l* mutants point to involvement of only PSY2L but not PP4R2L in certain processes important for growth and development.

### Genotoxicity assay

**Fig 4:**
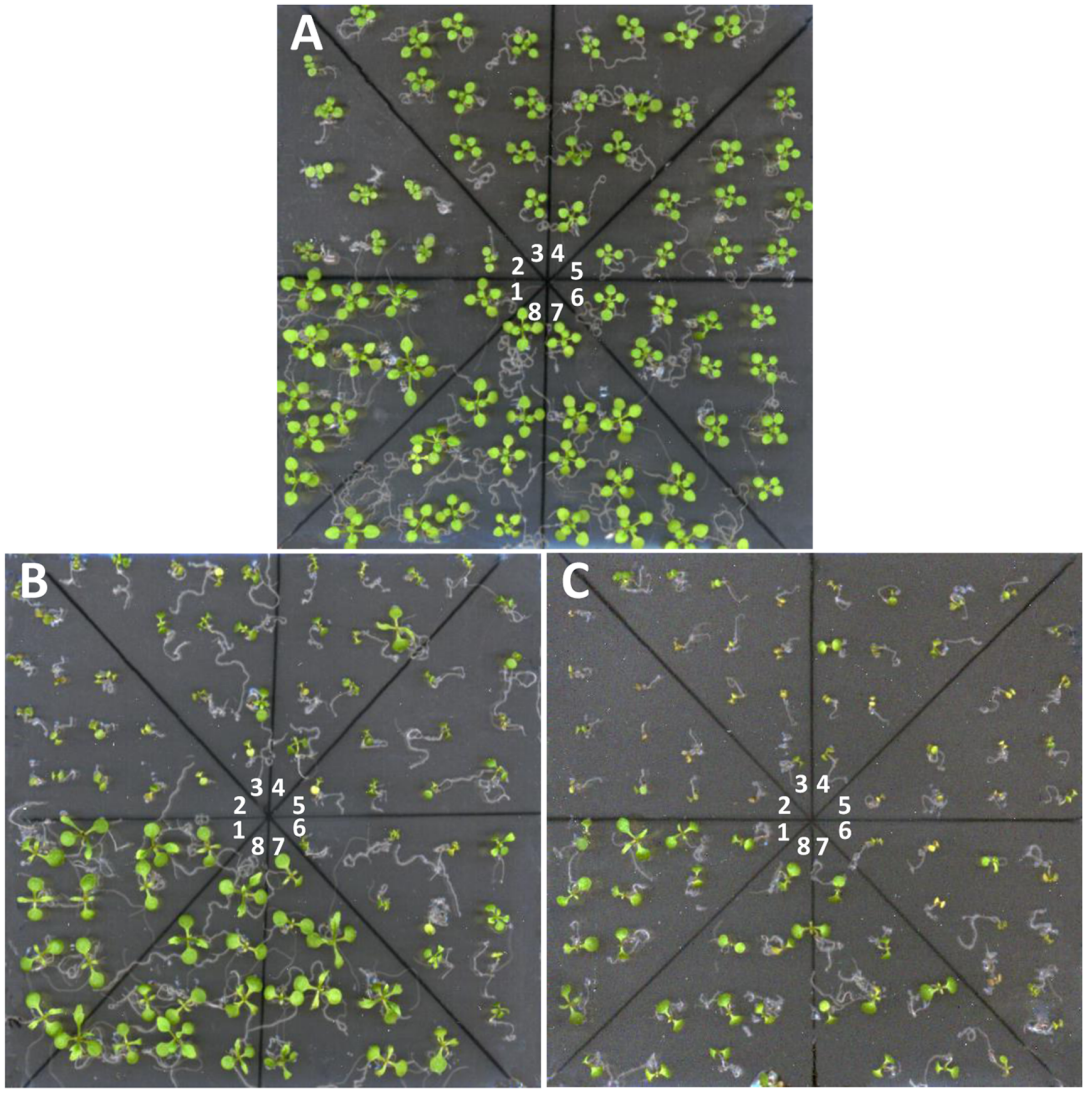
*PSY2L* knockout and knockdown mutants are hypersensitive to cisplatin. After growing three days on ½ MS medium with 1% sucrose seedlings were transferred to new media for another 12 d for treatments. **A**, no cisplatin. **B**, 2 mg L^-1^ cisplatin. **C**, 4 mg L^-1^ cisplatin. The different plant lines in each Petri dish were 1: EV/Col-0; 2: *psy2l*; 3: *psy-ami1-1*, 4: *psy-ami1-2*, 5: *psy-ami2-1*, 6: *psy-ami2-2*; 7: *pp4r2l-ami1*; 8: *pp4r2l-ami2*.

Three days old seedlings grown on ½ MS medium were transferred to new medium supplemented with 0–4 mgL^-1^ cisplatin and were allowed to grow horizontally for another 12 days (Fig 4, statistics in S7 Fig). When compared to cisplatin free media (Fig 4A), seedlings from *PSY2L* mutants (*psy2l*, *psy-ami1* (1, 2), *psy-ami2* (1, 2) showed severe growth retardation (Fig 4B) and less survival (Fig 4C) on media supplemented with cisplatin. Control WT and the knockout of *PP4R2L* (*pp4r2l-ami1* (1, 2)) behaved similarly and were far less influenced by cisplatin than *psy2l* and *ami-psy* (Fig 4A–4C). Higher cisplatin concentrations (6 and 8 mgL^-1^) were also tested, but strongly prevented growth in all plants (S7 Fig).

### PP4-1, PP4-2, PSY2L and PP4R2 target nucleus and cytosol

**Fig 5:**
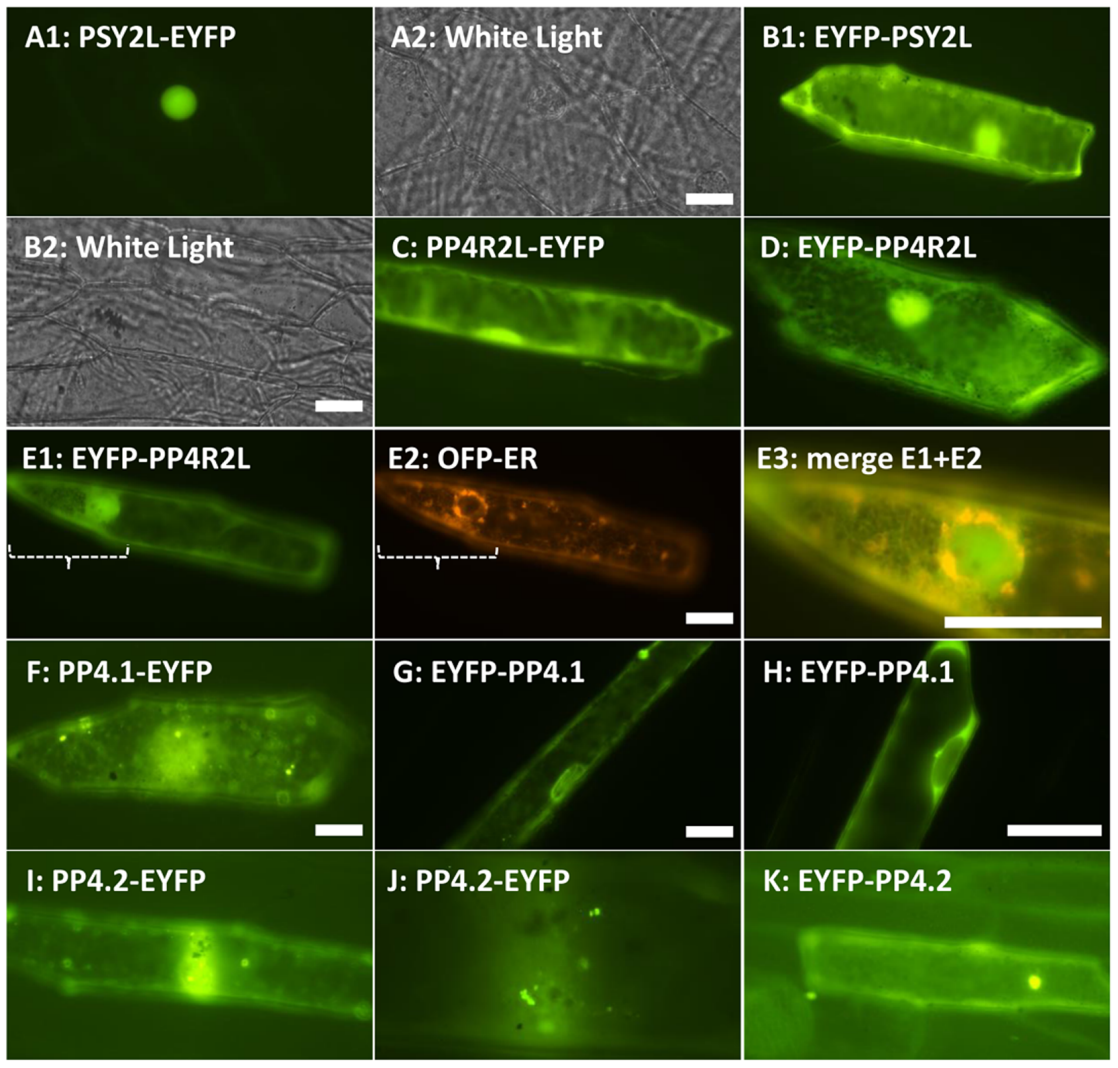
Subcellular targeting analysis for PP4 catalytic and regulatory subunits in onion epidermal cells. Fusion proteins were precipitated on gold, bombarded into onion epidermal cells, and examined after 16 h. **A**, PSY2L with free N-terminus targeted only nucleus. **B**, PSY2L with free C-terminus targeted nucleus and cytosol. **C-E**, PP4R2L targeted cytosol (C and D), nucleus (C-E) and endoplasmic reticulum (D, E). Partial overlap between OFP-ER and free C-terminus PP4R2L was detected in (E). **F-H**, PP4.1 showed a variability of targeting patterns including cytosol (F-H) and weak nucleus targeting (F, H) and unknown punctate structures (F). In addition, in some cells, also targeting of the nuclear envelope was seen (G, H). **I-K**, PP4.2 protein showed mostly targeting to cytosol, and unknown punctate structures (I-K). Endoplasmic reticulum was labeled by OFP-ER [25]. Scale bars = 20 μM.

All four genes were fused with enhanced yellow fluorescent gene (*EYFP*) to give both free N- and free C- terminus of the protein of interest. The gene constructs were expressed in onion epidermal cells (Fig 5) and Arabidopsis mesophyll protoplasts (S8 Fig). In both expression systems, PSY2L with free N-terminus targeted nucleus without cytosolic background (Fig 5A, S8A Fig), and with free C- terminus targeted both nucleus and cytosol (Fig 5B, S8B Fig). PP4R2L with free N-terminus targeted cytosol (Fig 5C, S8C Fig) and possibly nucleus (Fig 5C). PP4R2L with free C-terminus clearly targeted nucleus in addition to cytosol (Fig 5D). In addition, a partial ER-like network was detected for PP4R2L with free C-terminus (Fig 5E). The fusion proteins for PP4-1 had different targeting patterns depending on cells and expression systems. PP4-1 fusions showed cytosol and nucleus targeting (Fig 5F–5H, S8E–S8I Fig). Moreover, the PP4-1 fusion proteins were detected in unknown punctate structures (Fig 5F, S8F–S8H Fig), and a network like structure around nucleus in onion epidermal cells (Fig 5G and 5H). However, these structures did not coincide with ER in protoplasts (S8I Fig). Similar to PP4-1, PP4-2 fusions were detected in nucleus (S8I and S8K Fig), but mostly targeted to cytosol and unknown punctate structures in onion epidermis (Fig 5I–5K. S8J and S8K Fig). Altogether, the experiments indicate putative localization sites for PP4 in the cell. Localization patterns are complex and require further determinations of full PP4 complexes and also localization of the substrate(s) as they become known. In conclusion, all PP4 subunits were detected in the nucleus and in the cytosol, but with less frequency of PP4-2 in the nucleus.

### Global expression analysis of the *psy2l* mutant relative to WT

To find genes consistently influenced by *PSY2L*, two different tissue types were investigated.

**Fig 6:**
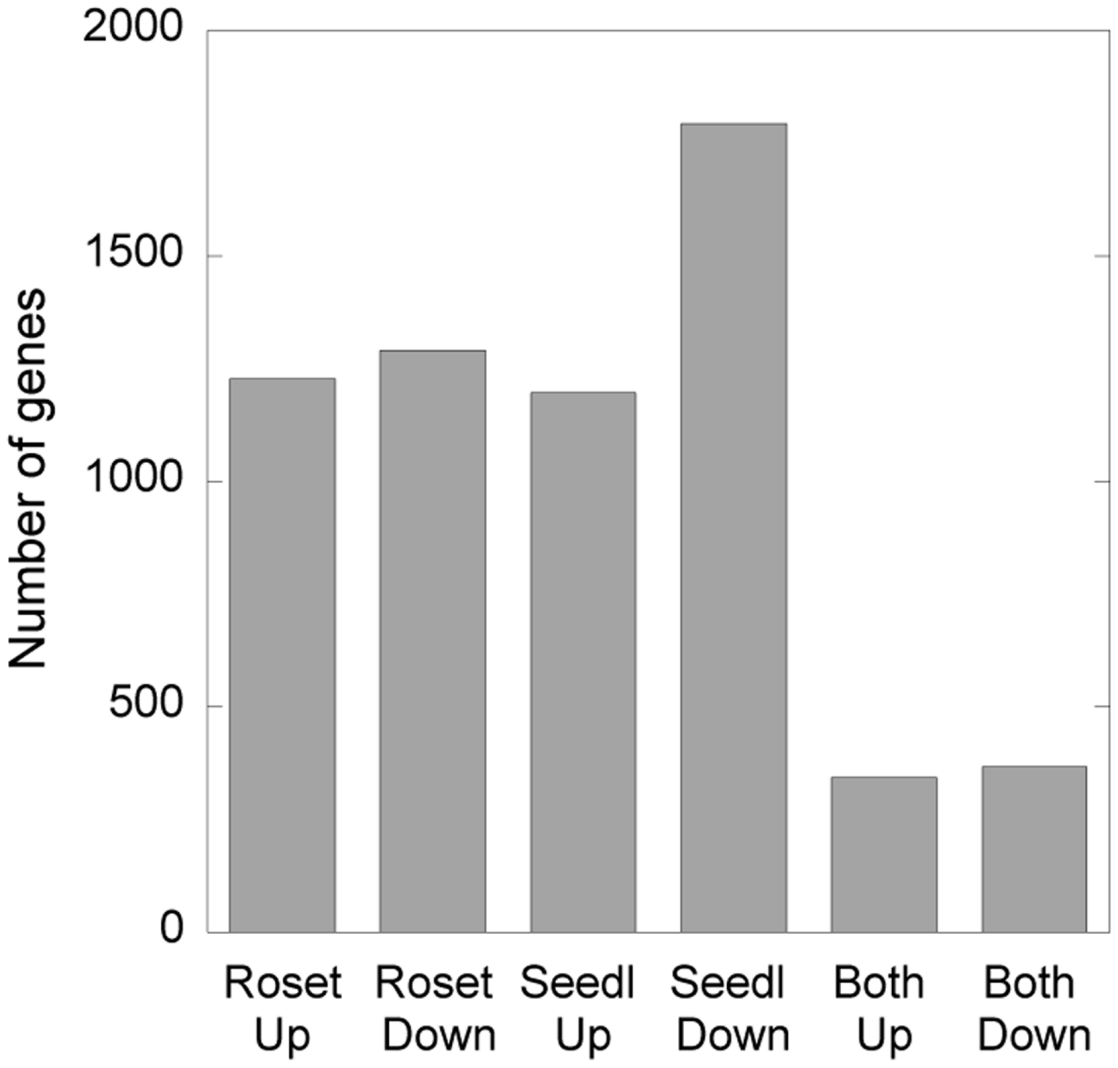
Number of genes at least two-fold higher or lower expressed in *psy2l* compared with WT. The two left columns represent rosette leaves from plants in soil, the two middle columns represent 8–10 days old seedlings, and the two columns to the right represent joint genes two-fold different from WT in both rosettes and seedlings. Number of genes are from expression data of three biological mutant samples versus three WT samples for each type of tissue, p<0.05 for genes listed as significant differently expressed (S1–S4 Tables).

Genes, 2517, differentially expressed in *psy2l* rosette leaves by factor 2 high or low compared with WT, were tested with the singular enrichment analysis (SEA) AgriGo bioinformatics tool kit [26]. Likewise, 2989 genes from *psy2l* seedlings were compared with WT seedlings (Fig 6, S1–S4 Tables). When examining “Molecular function” and “Biological Process” using the AgriGO tool, several groups of significantly enriched genes were delivered. Interesting GO terms related to the observed *psy2l* phenotype and significantly enriched are listed in Table 1. When examining “Cellular Component” with the AgriGo tool, “nucleus” was the clear cut significant subcellular compartment. Genes of different GO-terms were further compared for rosette leaves and seedlings to identify joint genes with expression similarly perturbed in the two different tissue types (Tables 1 and 2). Although PSY2L obviously may regulate different genes in specialized tissues, focusing on the genes coregulated in both tissues should help to identify specific genes most likely influenced by *PSY2L* (Table 2).

**Table 1:** Singular enrichment analysis (SEA) using AGriGO for genes more than two-fold differently expressed in *psy2l* versus WT. GO terms of special interest for the phenotype observed are presented. Genes were sorted as Up or Down- regulated. Non-significant is marked as *ns*. Input number for two-fold up-regulated genes were 1227 and 1196, and two-fold down-regulated were 1290 and 1793 genes for rosette leaves and seedlings, respectively. Number of genes in background reference (BG/Ref) is given for each GO term. Total annotated number in background reference was 31819 genes.

Description/GO term	Up or down-regulated	Rosette number of genes	Seedling number of genes	BG/ref	p-value Rosette	p-value Seedling	Shared genes
**MOLECULAR FUNCTION**							
**Kinase activity** GO:0016301	Up	37	57	417	8.00E-06	2.50E-15	12
	Down	93	83	417	5.20E-36	2.70E-20	21
Protein tyrosine kinase activity GO:0004713	Up	9	12	33	1.90E-05	4.60E-08	3
	Down	14	19	33	1.80E-09	8.40E-12	3
**Protein Ser/Thr phosphatase activity** GO:0004722	Up	15	12	58	6.20E-08	7.40E-06	4
	Down	ns	10	58	ns	3.10E-03	
**BIOLOGICAL PROCESS**							
**Anatomical structure and development** GO: 0048856	Up	74	70	330	2.60E-30	4.10E-28	22
	Down	35	67	330	1.10E-06	5.20E-17	9
**Response to hormone stimulus** GO:0009725	Up	63	64	755	4.90E-08	8.80E-09	18
	Down	68	85	755	5.90E-09	1.30E-08	18
Response to abscisic acid stimulus GO:0009737	Up	37	40	276	6.30E-10	7.40E-12	15
	Down	ns	ns	276	ns	ns	
Response to ethylene GO:0009723	Up	9	12	30	1.00E-05	2.00E-08	1
	Down	17	7	30	9.00E-13	3.10E-03	3
Response to gibberellin stimulus GO:0009739	Up	11	7	19	5.00E-09	2.90E-05	3
	Down	16	10	19	5.20E-14	1.40E-06	6
Response to cytokinin stimulus GO:0009735	Up	ns	ns	72	ns	ns	
	Down	11	15	72	3.70E-04	5.30E-05	5
**Flavonoid biosynthetic process** GO:0009813	Up	16	9	61	1.90E-08	9.30E-04	7
	Down	ns	ns	61	ns	ns	
**Transport** GO:0006810	Up	89	69	540	1.40E-27	6.40E-17	24
	Down	93	107	540	1.80E-28	1.10E-25	23
**CELLULAR COMPONENT**							
Nucleus GO:0005634	Up	107	99	212	7.2E-72	4.8E-65	31
	Down	74	98	212	1.4E-39	1.2E-48	18

**Table 2:** Relative expression levels of highly interesting selected genes. Data given are fold expression changes in *psy2l* mutant relative to WT. Down-regulation of genes in *psy2l* is indicated by a minus.

Arabidopsis ID	Gene	Description	Rosette versus WT	Seedling versus WT
**Kinase activity**				
AT3G16360	AHP4	Histidine-containing phosphotransmitter	9.30	4.42
AT1G66830	*AT1G66830*	Leucine-rich repeat protein kinase family protein	6.54	12.36
AT1G02970	WEE1	WEE1 KINASE HOMOLOG	2.34	2.16
AT3G45430	LECRK15	L-TYPE LECTIN RECEPTOR KINASE I	-8.29	-5.56
AT3G55950	CRINKLY3	CRINKLY4 related 3 (CCR3)	-6.81	-3.04
AT1G53700	WAG1	PROTEIN KINASE 3	-3.89	-2.88
**Protein Ser/Thr phosphatase**				
AT5G59220	HAI1	HIGHLY ABA-INDUCED PP2C GENE 1	18.25	2.41
AT1G07430	HAI2	HIGHLY ABA-INDUCED PP2C GENE 2	7.23	8.15
AT3G17790	PAP17	Purple acid phosphatase 17	2.46	2.38
AT4G26080	ABI	ABA INSENSITIVE 1	2.36	2.23
**Anatomical structure and development**				
AT3G17520	LEA	Late embryogenesis abundant protein (LEA) family protein	7.00	6.12
AT4G36600	LEA	Late embryogenesis abundant protein (LEA) family protein	4.09	6.34
At1G02205	CER1	Fatty acid hydroxylase	2.67	2.33
AT5G53200	TRY	Homeodomain-like superfamily protein	-23.96	-9.38
AT1G13950	ELF5	EUKARYOTIC ELONGATION FACTOR 5A-1	-5.67	-4.00
**Response to ABA**				
AT2G46680	ATHB7	ARABIDOPSIS THALIANA HOMEOBOX 7	11.35	2.63
AT2G46270	GBF3	G-BOX BINDING FACTOR 3	7.36	2.24
AT2G46270	ABI5	ABA INSENSITIVE 5	6.26	2.61
**Response to cytokinin**				
AT1G74890	ARR15	Two-component response regulator 15	-4.56	-9.06
AT3G48100	ARR5	Two-component response regulator 5	-3.11	-3.90
AT2G41310	ARR8	Two-component response regulator 8	-2.59	-2.46
**Response to GA**				
AT5G50915	BHLH137	Basic helix-loop-helix (bHLH) DNA-binding superfamily protein	-4.40	-7.87
**Flavonoid biosynthetic process**				
AT5G42800	DFR	DIHYDROFLAVONOL 4-REDUCTASE	54.3	22.08
AT1G56650	PAP1	PRODUCTION OF ANTHOCYANIN PIGMENT 1	41.13	14.74
AT4G09820	TT8	TRANSPARENT TESTA 8	34.9	7.59
AT4G22880	LDOX	LEUCOANTHOCYANIDIN DIOXYGENASE	31.44	13.87
AT3G29590	5MAT	Involved in the malonylation of anthocyanins	23.18	10.59
AT2G37260	TTG2	Encodes a protein similar to WRKY transcription factors	5.69	4.04
**Transport**				
AT1G77380	AAP3	AMINO ACID PERMEASE 3	8.55	6.22
AT1G61800	GPT2	GLUCOSE-6-PHOSPHATE/PHOSPHATE TRANSLOCATOR 2	7.38	7.90
AT2G38530	LTP2	LIPID TRANSFER PROTEIN 2	7.37	8.27
AT3G55110	ABCG18	ABC transporter	5.47	6.30
AT1G73700	*AT1G73700*	MATE efflux family protein	4.44	10.40
AT2G04040	DTX1	DETOXIFICATION 1	4.01	4.58
AT5G52050	DTX50	DETOXIFICATION EFFLUX CARRIER 50	-9.97	-4.13
AT5G26200	F9D12.12	Mitochondrial substrate carrier family protein	-4.05	-5.61
**Nucleus**				
AT1G13370	*AT1G13370*	Putative histone H3	17.65	4.89
AT3G09480	*AT3G09480*	Putative histone H2B	9.80	4.01
AT3G53730	*AT3G53730*	Histone superfamily protein with H4-site	7.14	3.26
AT3G45930	*AT3G45930*	Histone superfamily protein with H4-site	4.64	3.04
AT5G10980	HTR8	Histone H3.3, HTR8	4.43	2.70

#### Kinase activity genes

Kinase activity genes in *psy2l* constituted an enriched GO term (Table 1). Many of these genes, e.g. 93 in rosette leaves and 83 in seedlings, were down-regulated in the mutant relative to WT. From the two-fold down-regulated kinase genes, about half of them encoded protein kinases. Shared between both tissue types, 21 genes were more than two-fold down-regulated while 12 were up-regulated (Table 1). The putative receptor kinase *CRINKLY3* (AT3g55950) was lowered by factor 6.8 and 3.0 in the two tissue types (Table 2). Also other members of the small *CRINKLY4* gene family were down-regulated; *CRINKLY4* (AT5g47850) was down-regulated 24.9 times in *psy2l* rosette leaves (but not present in seedling gene list), and *CRINKLY1* (AT3g09780) was down-regulated 2.1 times in seedlings. The exception to down-regulation was *CRINKLY2* (AT2g39180), which was up-regulated 2.9 times in rosette leaves (but not present in seedling gene list). The last member of the group, *ACR4* (AT3g59420) was not present in lists for rosette leaves nor seedlings. The *CRINKLY4* group of receptor-like kinases is involved in a wide range of developmental processes, and down-regulation of *CRINKLY4* genes were found to give dwarf plants with misshapen leaves and low fertility [27, 28]. Altered expression of these genes in *psy2l* may contribute to the observed phenotype. Another protein kinase gene, *WAG1* (AT1g53700) was 3.9 times down-regulated in rosette leaves and 2.9 times down-regulated in seedlings. This protein kinase has a function in root development [29] and its strong down-regulation may be related to the effects seen in *psy2l*, e.g. poor root growth.

Among the 12 up-regulated kinase genes was the highly interesting *WEE1* gene, that is known to be transcriptionally activated by impaired DNA replication or by DNA damage [30]. *WEE1* was 2.4 times up-regulated in rosette leaves and 2.2 times up in seedlings (Table 2). The poor root growth observed in *psy2l* (Fig 2H–2M, S4 Fig) may be, at least partly, caused by up-regulation of *WEE1* in agreement with work by De Schutter et al. [30] where overexpression of *WEE1* led to arrest of root growth.

Tyrosine protein kinases were significantly enriched (Table 1), implicating *PSY2L* in regulation of these kinases. Many of the tyrosine protein kinases are annotated as localized to membranes, e.g. plasma membrane, endomembrane, or as transmembrane receptor proteins. Fourteen and 19 protein tyrosine kinase genes were two-fold down-regulated in rosette leaves and seedlings, respectively (only 3 shared). None of these kinases have been further characterized (TAIR database).

#### Protein serine/threonine phosphatase activity

Interestingly, kinases were generally more significantly enriched for down-regulated, while phosphatases were more enriched for up-regulated genes (Table 1). All the up-regulated phosphatases belonged to the PP2C group or PAPs (PURPLE ACID PHOSPHATASEs) with only one exception, a TOPP6 (PP1 type phosphatase) that was about two-fold up-regulated in *psy2l* seedlings. The PAPs have a broad range of substrates including both proteins and small organic compounds. They may have regulatory functions as well as functions in mobilization of phosphate [31, 32]. *PAP17* was up-regulated 2.4-fold in both types of *psy2l* tissues tested, and this phosphatase has been shown to display peroxidase activity [33]. Interestingly, the PP2C phosphatase *ABI1* (*ABA INSENSITIVE 1*) was up-regulated about 2.3 times in both tissue types. *ABI1* is known as a negative regulator of ABA promoting stomata closure [34, 35]. *HAI1* (*HIGHLY ABA-INDUCED PP2C GENE 1*) was up-regulated by factor 18.2 in rosette leaves and by factor 2.4 in seedlings. This gene is also annotated as a negative regulator of osmotic stress and ABA signaling. A close homolog, *HAI2*, was induced by factor 7–8 in both tissue types (Table 2).

#### Anatomical structure development

Taking into account the strikingly altered anatomy of *psy2l*, we analyzed the GO term “Anatomical structure development”. For the rosette leaves there were more up-regulated than down-regulated genes, whereas for seedlings the numbers were similar. For joint up-regulated genes (22 genes), many were flavonoid pathway or other epidermis-related genes, for example *CER1* (*ECERIFERUM 1*), a fatty acid hydroxylase related to production of stem epicuticular wax and pollen fertility [36]. These genes point to involvement of PSY2L in regulation of epidermis characteristics. Four LEA (LATE EMBRYOGENES GENES) genes with unknown function were more than four times up-regulated in both tissue types. LEA proteins appear to contribute to drought resistance during the vegetative stage, but most LEA genes have not been functionally characterized [37, 38]. *PSY2L* appears necessary to restrain expression of some *LEA* genes indicating a negative control by *PSY2L* on these *LEA* genes either as a secondary or as a more direct effect.

Only 9 genes were jointly down-regulated in the “anatomical structure development” GO term. *TRY*, a gene encoding a small MYB/homeodomain-like superfamily transcription factor involved in trichome distribution [39], was strikingly down-regulated. Knockout of *TRY* is known to give clustering of trichomes [39], but trichomes were evenly distributed on the leaves of the *psy2l* mutant. Another striking down-regulated gene was *ELF5A-1* (*EUKARYOTIC ELONGATION FACTOR 5A-1*) translation initiation factor. This is a conserved translation factor involved in promotion of ribosomal function. Expression of *ELF5A-1* was strongly down-regulated in both rosette leaves (5.7-fold) and seedlings (4-fold). This may be a key gene related to the slow growth phenotype of *psy2l*, and suppression of this gene is known to impair xylem formation [40].

#### Response to hormone stimulus—ABA

Hormones are likely to play important roles in forming the phenotype of *psy2l*, and sub-terms of the highly significantly enriched GO term: “Response to hormone stimulus” were inspected (Table 1). Up-regulated, but not down-regulated, ABA-stimulated genes constituted an enriched group. Among the up-regulated genes, 15 genes were common for both rosette leaves and seedlings (Table 1). Five of these shared genes were transcription factors, and most strikingly up-regulated were *ATHB7* and *ATHB12* (*HOMEOBOX 7* and *12*) (Table 2). Especially *ATHB7* was up-regulated from 23 to 262 FPKM (fragment per kilobase per million mapped reads) in rosette leaves and from 27 to 72 FPKM in seedlings. Recently, high expression level of *ATHB7* was found to delay senescence in Arabidopsis [41]. Delayed senescence (longevity) was a striking phenotypic trait in *psy2l* plants. Since high expression of *ATBH7* was also pronounced in seedlings, this supports *PSY2L* being a suppressor of *ATBH7*. Interestingly, *ABI5* was up-regulated in *psy2l*. *ABI5* is known as an inhibitor of germination [42], hence high expression of *ABI5* is relevant in relation to the delayed germination observed for *psy2l* (Fig 3B).

#### Response to hormone stimulus—Ethylene

“Response to ethylene” was a significantly enriched gene group (Table 1), however, only one shared gene was more than two-fold up-regulated in both rosette leaves and seedlings. This was the *MYB13* gene also found in the ABA responsive group of genes. Three ethylene responsive genes, all transcription factors, were more than 3-times down-regulated. Most strongly influenced was *ERF15* (*ETHYLENE-RESPONSIVE ELEMENT BINDING FACTOR 15*), which was 7 and 3 times down-regulated in rosette leaves and seedlings, respectively. *ERF15* was recently found as a positive regulator of ABA response [43].

#### Response to hormone stimulus—Cytokinin

Down-regulated genes were enriched for stimulus to cytokinin in both rosette leaves and seedlings. In rosette leaves 5 of these down-regulated genes were two-component response regulators, and in seedlings 7 of these genes were two-components response regulators. *ARR15* (*RESPONSE REGULATOR 15*) and *ARR5* (*RESPONSE REGULATOR 5*) were strongly down-regulated, 5–9 times for *ARR15* and 3–4 times for *ARR5* (Table 2). Both *ARR15* and *ARR5* are known to be induced or stabilized by cytokinin (TAIR annotation) indicating that the cytokinin level in the *psy2l* mutant is lowered relative to WT.

#### Response to hormone stimulus—Gibberellic acid and auxin

The GO term “Response to gibberellin stimulus” was highly enriched for down-regulated genes, and six genes were common to rosette leaves and seedlings (Table 1). Five of the 6 genes were transcription factors, three genes were also responsive to auxin, according to GO annotation. The most striking gene was *BHLH137* (At5g50915), which was 4.4 and 7.9 times down-regulated in rosette leaves and seedlings, respectively (Table 2). The “response to auxin” GO term gave variable results for rosette leaves and seedlings with down-regulated genes being highly significant for rosette leaves, but not significant for seedlings. However, when specifically searching for *SAUR* (SMALL AUXIN UP RNAs) genes, two-fold changed, 13 down-regulated *SAURs* were found for rosette leaves (and one up-regulated), and eleven down-regulated *SAUR* genes were found for seedlings (and two up-regulated) (data in S1–S4 Tables). Since *SAUR* genes are markers for auxin effects [44], these results point to auxin levels as being lower in the *psy2l* mutant in comparison with WT.

#### Flavonoid biosynthetic process

The *psy2l* mutant easily developed purple colored leaves although control WT plants in the same pots did not (Figs 2D and 3A). This was reflected in the up-regulation of several genes involved in flavonoid synthesis (Tables 1 and 2). Transcripts of the general flavonoid pathway regulator *MYB75/PAP1* (*PRODUCTION OF ANTHOCYANIN PIGMENT 1)* was up-regulated 41 and 15 times in rosette leaves and seedlings, to a high expression level e.g. 55.9 and 54.5 FPKM, respectively. A bHLH transcription factor promoting the last steps in proanthocyanin and anthocyanin synthesis, *TT8 (TRANSPARENT TESTA 8)* was also strongly induced, e.g. 8–35 times, resulting in FPKM levels around 10 for both seedlings and rosette leaves. On the other hand, the *TT8* close homologs *GL3* and *EGL3* which usually stimulates anthocyanin synthesis in Arabidopsis leaves [22], were expressed only at a low level. The regulator of *TT8*, *TTG2/WRKY44* was also expressed at a high level, 4 and 6 times higher in *psy2l* seedlings and rosette leaves, respectively, compared with WT. Structural genes of the anthocyanin branch of the flavonoid pathway are positively regulated by PAP1 and TT8 in complex with the constitutive TTG1 protein [45], and this is in line with *DFR* (*DIHYDROFLAVONOL 4-REDUCTASE*) and *LDOX* (*LEUCOANTHOCYANIDIN DIOXYGENASE*) expression being enhanced 14–54 times in *psy2l* rosette leaves and seedlings (Table 2).

#### Transport

The GO term “Transport” was highly enriched (Table 1). In common for rosette leaves and seedlings 24 genes were up-regulated and 23 genes were down-regulated (Table 1). The affected genes included all kinds of different transporters, like MATE (multidrug transporters), ABC (ATPase coupled transporters), POT (proton-dependent oligopeptide transporters), transporters involved in iron, phosphate, sulfate, ammonium, lipid, purine and sugar transport. Genes co-regulated in both tissue types and more than 4-fold perturbed in comparison with WT are listed in Table 2. Transporters implicated with lipid transport were highly represented in both up and down-regulated genes, e. g. a total of 10 joint genes. In down-regulated genes, the presence of 5 chloroplast and two mitochondrium transporters indicates that functions in these organelles, are influenced by *PSY2L*. A mitochondrial inner membrane carrier (At5g26200) was down-regulated 4–6 times in both rosette leaves and seedlings (Table 2). Related to chloroplasts, a gene involved in protein folding and transport (At2g30695), containing a conserved domain, bacterial ribosome binding trigger factor, was down-regulated 2.4 and 2.9 times, but appeared very stable in WT control tissue. A chloroplast envelope sugar/phosphate antiporter gene, *GPT2* (Glucose-6-phosphate/Pi transporter), was up-regulated by factor 7.4 and 7.9 in rosette leaves and seedlings. GPT2 allows equilibration of glucose-6-phosphate and phosphate in the cell. GPT2 is induced by high sugar levels and in response to various other endogenous and external signals [46]. The data are compatible with *PSY2L* as a suppressor of *GPT2*.

#### Nucleus

“Nucleus” was the most highly enriched “subcellular compartment” GO term with 49 genes jointly up or down-regulated in rosette leaves and seedlings. These were mainly transcription factors, e.g. 31 genes, many already mentioned as influenced by hormones.

Most striking was a group of 8 histones, all up-regulated (At1g13370, At2g28720, At2g28740, At3g09480, At3g45930, At3g46320, At3g53730, At5g10980). Most were highly up-regulated, e.g. 4–17 times (Table 2). The physiological significance of this up-regulation is not clear, but changes in histone composition are involved in cell cycle progression in Arabidopsis [47, 48].

#### DNA damage repair response and cell cycle arrest

We inspected expression of genes conserved in eukaryotes and involved in DNA double strand break (DSB) repair (genes listed in: Amiard et al. [49]). This revealed 19 genes with changed expression in *psy2l* versus WT (Table 3). Additionally, 11 DNA repair genes were selected by the AgriGo tool. Several of the DNA repair associated genes are induced by radiation, like *BRAC1*, *GR1*, *XRI1*, *RAD17*, *RAD51* and *RAD54*. The lower part of Table 3, with AgriGo tool selected genes, comprises also DNA repair genes not involved in DSB repair (DNA glycosylases). Furthermore a gene not revealed by the Amiard list or AgriGO, e.g. *WEE1*, was up-regulated in *psy2l* and is also considered important for DSB repair in Arabidopsis [24] (Table 2). DNA damage repair signaling and cell cycle arrest are tightly connected [30, 50]. DNA damage activates signaling pathways through the sensor kinases ATM and ATR and the signaling will activate cell cycle arrest that allows time for DNA repair [49]. In Arabidopsis, the cell cycle inhibitor kinase WEE1 is transcriptionally activated in response to DNA damage or cessation of DNA replication signaled through ATR [30]. Furthermore, the cell cycle inhibitors and checkpoint regulators SMR5 and SMR7 are known to be transcriptionally activated by genotoxic stress [50]. These genes were also up-regulated in the *psy2l* mutant. *SMR7* was 7-fold up in both *psy2* rosette leaves and seedlings (S1 and S3 Tables), strongly indicating that cell cycle progress was impaired.

**Table 3:** Genes involved in DNA double strand break signaling and repair in Arabidopsis. Listed according to Amiard et al. (2013) [49]. Additional genes involved in DNA repair identified using AgriGo (Go Analysis Toolkit and Database for Agricultural Community) [26] are added. Arabidopsis ID numbers marked with * are involved in DNA double strand break repair according to AgriGO SEA or TAIR.

Function	Arabidopsis ID	Gene	Description	Rosette versus WT	Seedling versus WT
**Sensing**					
	AT2G31970*	*RAD50*	Encodes the Arabidopsis RAD50 homolog		-1.45
Signaling					
	AT1G08880	*H2AX*	Encodes HTA5, a histone H2A protein.	1.98	
	AT1G54690	*H2AX*	Encodes HTA3, a histone H2A protein.	1.87	
**Mediators**					
ATM signaling					
	AT4G21070	*BRCA1*	Encodes AtBRCA1, an orthologue of the human breast cancer susceptibility gene 1	1.93	2.37
	AT3G52115*	*COM1/GR1*	GAMMA RESPONSE GENE 1	2.46	2.11
ATR signaling					
	AT3G05480	*RAD9*	Cell cycle checkpoint control protein family	2.36	
	AT5G45400	*RPA1C*	REPLICATION PROTEIN A 1C		1.94
	AT4G19130	*RPA1E*	Replication factor-A protein 1-related		2.88
HR					
	AT5G20850*	*RAD51*	Encodes a homolog of yeast RAD51	4.31	1.77
	AT2G28560*	*RAD51B*	Encodes a protein of the RAD51B	-4.48	
	AT2G45280*	*RAD51C*	Encodes a protein similar to RAD51C	1.62	
	AT5G64520*	*XRCC2*	Encodes a protein of the XRCC2 family	2.24	
	AT5G57450*	*XRCC3*	Homolog of X-RAY REPAIR CROSS COMPLEMENTING 3		2.78
	AT1G71310*	*RAD52-1*	RADIATION SENSITIVE 51–1		1.37
	AT3G05210*	*ERCC1*	Encodes a homolog of human ERCC1 protein (yeast RAD10)		-1.33
NHEJ					
	AT1G48050*	*KU80*	Arabidopsis thaliana KU80 homolog	1.35	-1.38
	AT1G80420	*XRCC1*	HOMOLOG OF X-RAY REPAIR CROSS COMPLEMENTING 1.		-1.31
	AT2G31320	*PARP1*	Encodes a poly(ADP-ribose) polymerase	1.90	1.54
	AT4G02390*	*PARP2*	POLY(ADP-RIBOSE) POLYMERASE 2	1.82	1.85
**Additional DNA-repair genes, variable functions**					
	AT3G48425	*DNAse I-like*	DNAse I-like superfamily protein.	-2.16	
	AT3G22880	*DMC1*	DISRUPTION OF MEIOTIC CONTROL 1	3.28	2.64
	AT5G40840*	*SYN2*	SISTER CHROMATID COHESION 1 (SCC1) PROTEIN HOMOLOG 2	2.82	2.54
	AT5G44680	*DNA glycosylase*	DNA glycosylase superfamily protein		-2.36
	AT5G66130	*ATRAD17*	Encodes a homolog to yeast RAD17	2.81	4.62
	AT3G12710	*DNA glycosylase*	DNA glycosylase superfamily protein	-2.82	
	AT4G29170*	*ATMND1*	Homolog of yeast, mouse and human mnd1		1.60
	AT3G47830	*DNA glycosylase*	DNA glycosylase superfamily protein	-2.29	
	AT5G48720	*XRI1*	Encodes XRI1 (X-ray induced 1)	2.10	3.72
	AT3G19210*	*ATRAD54*	Encodes RAD54, member of the SWI2/SNF2 family of DNA-stimulated ATPases	2.42	
	AT5G54090	*MutS*	DNA mismatch repair protein MutS, type 2	6.90	

## Discussion

### Kinases and phosphatases

Generally, protein phosphatases inactivate protein kinases by dephosphorylation of the activation loop in kinases, and additional sites may also be regulated by phosphorylation/dephosphorylation. Hence, when a crucial protein phosphatase is impaired this may lead to increased phosphorylation status of certain protein kinases, which may further lead to induction of a negative feedback on gene expression to restore normal levels of kinase activity. This may be part of the explanation for enrichment of down-regulated kinase genes (Table 1). Furthermore, impairing the activity of an important phosphatase complex like PP4c-PSY2L may lead to enhanced expression of other protein phosphatases as an attempt to establish homeostasis by up-regulation of phosphatases that partly can replace the impaired phosphatase. Up-regulated protein phosphatase genes were enriched, especially *PP2C* and *PAP* phosphatases.

### Flavonoids

Typical nutrient stress sensitive regulators of the flavonoid/anthocyanin pathway, *PAP2* and *GL3* [22], were not influenced by *PSY2L* knockout, but expressed at a very low level in both seedlings and rosette leaves, as in WT. Furthermore, the *HY5* gene which acts as an integrator of light signaling for promoting flavonoid syntheses was not consistently up-regulated, but was increased by factor 1.8 in seedlings and decreased by factor 0.6 in rosette leaves (S6 and S7 Tables). The *TT8* gene is generally highly expressed in developing seeds, and not induced by stress factors like nutrient depletion or high light intensity (TAIR database, eFP Browser and [9, 45, 51]. The strong upregulation of *TT8* expression in both *psy2l* seedlings and rosette leaves is intriguing (Table 2). Apparently *TT8* has overtaken the function of its homologs *GL3* and *EGL3* that usually are important for anthocyanin synthesis in leaves [51]. Possibly, a phosphorylated regulator in the *psy2l* mutant, otherwise inactivated by dephosphorylation when PSY2L is present, may activate expression of *TT8*, *PAP1*, and *TTG2* in *psy2l*. All taken together *PSY2L* appears to act as an upstream, negative regulator of specific transcription factors, e.g. *PAP1* and *TT8*. High expression levels of these transcription factors explain the high levels of structural anthocyanin synthesis genes and accumulation of anthocyanins in *psy2l*.

### Sugar metabolism, a conserved PSY2 regulated function?

In yeast, mammals, and *C*. *elegans*, PSY2 has various regulatory roles regarding sugar transport and metabolism, including transport of glucose in yeast [16]. Many transporter activity genes showed altered expression in *psy2l*, and intriguingly the *GPT2* (glucose-6-P/phosphate) transporter was transcribed at a highly increased level in *psy2l*, e.g. 7–8 fold increased (Table 2). In WT, the GPT2 transporter appears to be generally repressed unless certain signals from environmental or developmental cues occur [46]. The results here point to *PSY2L* as a negative regulator up-stream of *GPT2*. When *PSY2L* is impaired, *GPT2* is constitutively expressed at a high level in very different tissues. Apparently, in a wide range of different eukaryotes PSY2L is involved in regulation of sugar transport and/or metabolism.

### Anatomical structures

The strikingly slow root growth, root hairs close to the root tip, and rippled morphology of the *psy2l* roots (Fig 2H–2M) resembles the phenotype found for WT roots treated with bleomycin to induce DNA double strand break [24]. Staining of the roots revealed less DNA in cells at the root tip, and a high number of dead cells (S4 Fig). The observed phenotype appears to be caused by impaired cell cycle progression, which can be induced by DNA repair signaling. RNA-seq data showed up-regulation for *WEE1*, *SMR5* and *SMR7*, all known to be transcriptionally up-regulated by DNA damage stress and to inhibit cell cycle progression [30, 50]. Ectopic expression of *SMR5* and especially *SMR7* hampered cell division and growth of shoots [50]. Also for *psy2l*, growth beyond the seedling stage, including pollen formation and seed set, is likely hampered by restricted cell cycle progression. Overall, the phenotype and expression analysis strongly underpins that PSY2L has a function in control of cell cycle progression.

PSY2L may have several targets, and targets other than the cell cycle for explaining the phenotype should not be excluded. In other multicellular organisms *PSY2* was also important for growth and development. In Drosophila, *PSY2* (falafel) knockout disturbed physiological development, i.e. special tissues, eyes and wings, started to die [52]. Overexpression of *PSY2* (*SMK1*) in *C*. *elegans* resulted in worms that could not be maintained as stable lines, and the F1 progeny died during embryogenesis [13]. In the work presented here, expression analysis showed that several genes annotated as involved in anatomical structure development showed altered expression levels in the *psy2l* mutant. Interestingly, both transcription factors *HOMEOBOX 7* (*ATHB7*) and *HOMEOBOX12* transcripts were significantly up-regulated. A PP4c-PSY2L complex may act as an upstream negative regulator of such transcription factors in Arabidopsis.

There are three ELF5/eIF5 (EUKARYOTIC ELONGATION FACTOR 5A) translation initiations factors in Arabidopsis, and ELF5A1 has a special function in formation of the xylem [40]. It was previously shown by Liu et al. [40] that mutants with overexpression of *ELF5-1* had a thicker layer of xylem cells and thicker flowering stems, while reducing the level of ELF5-1 to 50% of WT levels resulted in thinner layers of xylem and reduced radius of the flowering stems. In our study, expression of *ELF5-1* was reduced to about 20% of WT levels in both seedlings and rosette leaves. The flowering stems of the *psy2l* mutant often appeared flimsy and not able to stand upright like in WT. Possibly *PSY2L* may have a direct effect on transcription factors regulating *ELF5-1* expression or alternatively a more indirect effect through influencing hormone levels.

The *psy2l* phenotype with delayed germination (Fig 3) and impaired growth (Fig 2) was consistent with the expression data, which indicated high ABA, but low GA, cytokinin and auxin levels. Interestingly, it was recently also reported that genotoxic stress induced DNA repair signaling and delayed germination in a *SMR5* dependent manner [53].

### DNA damage checkpoint and cell division

In addition to cytosol and nucleus, PP4c is known to localize to centrosome/spindle pole bodies in human and Drosophila cells [54, 55]. Centrosome/spindle pole bodies are parts from microtubule-organizing centres (MTOCs) that are responsible for meiotic and mitotic spindle apparatus organization during cell division. Plants do not have centrosome/spindle pole bodies, and the nuclear envelope is thought to play the role of microtubule-organizing centres [56]. In this study, one of the obtained targeting patterns for tagged Arabidopsis PP4c is a network-like structures around nucleus (Fig 5G and 5H). These structures may be of interest for future investigation to study the role of PP4 in cell division in plants. In mammals and yeast phosphorylated H2AX histones is an important signal for DNA damage, and PP4 dephosphorylation of the H2AX histone is required for recovery from the DNA damage checkpoint [57]. In contrast, phosphorylation/dephosphorylation of this histone does not seem to be important in Arabidopsis. It still needs to be clarified if other histones have such a function in plants [49]. The hampered growth of *psy2l* roots and aberrant meristem, sensitivity to cisplatin, and up-regulation of DNA damage and cell cycle arrest genes substantiate the involvement of Arabidopsis PSY2L in maintenance of genome integrity. The connection of plant PP4 with the DNA damage checkpoint deserves further investigation.

### Conclusion

Although some chains of events seem straightforward, like high *TT8* and *PAP1* expression causing high levels of anthocyanins, some caution should also be taken regarding interpretation of transcript levels as markers for stimulation versus inhibition of a biological process. High transcript level of a gene may sometimes reflect that the translated product is not functional and a negative feedback loop could thereby have been distorted. This could be caused by lack of dephosphorylation of a protein by PP4c-PSY2L. Gene expression analysis can give only indications of which pathways are influenced by PP4c-PSY2L since the primary action of PP4 complexes is dephosphorylation, which takes place on the protein level. The present work reveals *PSY2L* as an essential regulator for growth and development in plants, likely implicating DNA damage signaling and cell cycle progress. Several perturbed genes and pathways have been identified, and these data pave the way for further exploration of the involvement of PSY2L in specific physiological processes, tissue types, and interaction with candidate genes and proteins.

## Materials and methods

### Plant material

Arabidopsis T-DNA insertion lines GK_651B07, SALK_070977 for *PP4-1*; SAIL_569_H09, SALK_049725C for *PP4-2*; SALK_048064, SALK_125872, SAIL_1275_F05, SAIL_33_H01, SAIL_256_C08 for *PSY2L*; and SALK_093041 for *PP4R2L* were obtained from the European Arabidopsis Stock Centre (Nottingham, UK) [58–60]. Screening for homozygous T-DNA insertions was accomplished by PCR using primers (see S5 Table) for T-DNA insertion lines recommended at the Salk Institute Web Site Signal (http://signal.salk.edu/tdnaprimers.2.html). Surface sterilized seeds were sown on agar containing half strength Murashige and Skoog (1/2 MS) medium [61], supplemented with 1% sucrose. Plates were placed at 4°C in the dark for two-three days, and were subsequently transferred to 16 h /8 h, or 12 h /12 h light/dark cycles as mentioned. Alternatively, plants were grown in soil supplied with Hoagland solution (15 mM KNO_3_) [62]. Generally the unpaired t-test was used to analyze the data.

### Generation of amiRNA and gene overexpressing transgenes

For construction of amiRNA expressing transgenes, we searched for potential targets against *PP4-1* and *PP4-2* (joint), *PP4R2L*, and *PSY2L* using default settings of the Web MicroRNA Designer (WMD) application (http://wmd3.weigelworld.org), based on the previously established parameters by [63, 64]. Mostly, the amiRNAs on the top of the provided list were chosen, and checked using the mirU [65] or the psRNATarget websites (http://plantgrn.noble.org/psRNATarget) [66]. Two potential amiRNAs were selected for each target, and their primers (I-IV, see S5 Table) were provided by the WMD3 website. Using these primers and two template specific primers (A and B, see S5 Table) PCR amplifications composed of two rounds were performed using the template plasmid pRS300 (Addgene: 22846) containing the miR319a precursor [64]. The amplified amiRNA transgenes were cloned in pGEMT-easy vector (Promega) and verified by sequencing. Subsequently, the transgenes were excised and subcloned into the 35S promoter-containing binary vector pBA002 [67]. In order to generate overexpressor lines of the selected genes, cDNAs were amplified and subsequently cloned into pBA002 vector.

The freeze-thaw method was used to transform the constructs into Agrobacterium ABI-1, which is a derivative of GV3101 (pMP90RK) and possesses the RK2 replicase and the trf gene required for plasmid replication. Hence, they were used for plant transformation using the floral dip method [68]. Screening of first to third generation seeds was performed on 1/2 MS agar plates containing 10 μg mL^-1^ phosphinothricin. Resistant seedlings were selected 10–14 d after germination.

### Cloning for subcellular localization

Templates used for amplification of Arabidopsis cDNAs for PSY2L (At3g06670), PP4R2L (Atg517070), and PP4-2 were U21916, U2491, U83558, respectively. The templates were obtained from the Arabidopsis Biological Resource center at Ohio State University (ABRC, Ohio). PP4-1 cDNA was amplified from the Wassilewskija PP4-1 cloned in pGEMT-vector kindly provided by prof. Jose J Sánchez-Serrano (Centro Nacional de Biotecnología, CSIC, Madrid, Spain). The amplified cDNAs were subcloned into pGEMT-easy (Promega, Madison, WI, USA), pCAT-EYFP [69, 70], and pWEN-EYFP [71] vectors in order to create N-terminal and C-terminal protein fusions with enhanced yellow fluorescent protein (EYFP). Subcloning vectors contain a 35S promoter of cauliflower mosaic virus. Details about primers and restriction enzymes are found in S5 Table.

### Subcellular localization and microscopy

Three to four weeks old plants grown in soil, at 12 h light/12 h darkness were used for protoplast isolation. Arabidopsis mesophyll protoplasts isolation and their subsequent PEG-transfection with plasmids were adapted after Sheen [72] and Yoo et al. [73]. Briefly, strips of Arabidopsis leaves were incubated with enzyme solution over-night at room temperature in the dark. The released protoplasts were filtered, centrifuged, and re-suspended in W5 solution. After 1 h incubation on ice, protoplasts were pelleted and re-suspended in MMg solution. The re-suspended protoplasts were subsequently transfected, using polyethylene glycol, with the above-mentioned plasmids, and incubated for 18 h-48 h. For transformation into onion epidermal cells, plasmids were precipitated onto gold particles, and transiently introduced by a helium-driven particle accelerator (PDS/1000; Bio-Rad, Hercules, CA, USA) with adjustments set to the manufacturer’s recommendations. The bombarded epidermal cell layer was incubated for one to two days. Transfected protoplasts and onion epidermal cells were then examined using fluorescence and confocal microscopes. Microscopy analysis was done using Nikon TE-2000U inverted fluorescence microscope equipped with an Exfo X-Cite 120 fluorescence illumination system and filters for YFP (exciter HQ500/20, emitter S535/30), Texas red filter set for RFP or OFP: 31004 (exciter D560/409, emitter D630/60 m), and a special red chlorophyll autofluorescence filter set (exciter HQ630/39, emitter HQ680/40; Chroma Technologies). Images were captured using a Hamamatsu Orca ER 1394 cooled CCD camera. The NIS-Elements AR analyses software (NIKON) was used to capture 0.5 Z-sections to generate extended focus images. Nikon A1R confocal laser scanning microscope using a 960 water objective was also used. Fluorescence images of EYFP (exciter 488, emitter 525), and OFP (exciter 561, emitter 595) were acquired and analyzed using the NIS-Elements AR analyses software (NIKON). Images were subsequently processed for optimal presentation with Adobe Photoshop version 9.0 (Adobe Systems, San Jose, CA, USA).

### Anthocyanin determination

Anthocyanin determination was adapted from Feyissa et al. [22]. Leaf tissue (0.05 g) was extracted in 300 μL extraction buffer consisting of 1% v/v HCl (1.2 M) in methanol. The leaves were extracted by constant shaking overnight at 4°C. Distilled water (200 μL) and chloroform (500 μL) were added and centrifuged at 13,000 x g for 2 min. The upper layer (400 μL) was added to an Eppendorf tube and mixed with 600 μL extraction buffer followed by centrifugation at two min at 13,000 x g. The absorbance was detected at 530 and 657 nm, and the relative concentration of anthocyanin was calculated as Abs _530_—Abs _657_.

### Alexander staining

Pollen viability was checked using Alexander’s stain [23]. Flowers that are about to open were dissected and dehiscent anthers were incubated with the stain on a microscope slide.

### Genotoxicity assay

Seeds were sown on ½ MS media (M5519, Sigma-Aldrich, St Lois, MO, USA) supplemented with 1% sucrose and 0.8% plant agar (Duchefa Biochemie, Haarlem, Netherlands), stratified for 2 d, and allowed to grow under 16 h light/8 h dark cycles. In order to prepare a stock solution of 0.5 mg/mL, cisplatin (cis-diamminedichloroplatinum (II), Sigma-Aldrich, St Lois, MO, USA) was dissolved primarily in 1 mL of dimethylformamide and mixed with 19 mL of 0.9% saline solution. In order to evaluate genotoxicity in control and mutant plants, 3 d old seedlings were transferred to media supplemented with 0–8 mg L^-1^ cisplatin and Petri dishes were placed horizontally for 12 d, or vertically for 3 d when investigating the effect on shoot and primary root development, respectively. Root measurements were accomplished using Image J (https://imagej.nih.gov/ij/index.html).

### qRT-PCR

For qRT-PCR, total RNA was extracted using RNAeasy Plant Mini Kit and treated with on-column DNaseI digestion (Qiagen, Hilden, Germany). One μg RNA was reverse-transcribed using the High Capacity cDNA Archive Kit (Applied Biosystems, Foster City, CA, USA) to generate first-strand cDNA in a 20 μL reaction volume. Quantitative real time PCR was performed on a Light Cycler 96 Sequence Detection System (Roche Diagnostics, Mannheim, Germany) using 96-well plates with a 15 μL reaction volume containing 7.5 μL of TaqMan buffer (Applied Biosystems; includes 6-Carboxyl-X-Rhodamine as a passive reference dye), 0.75 μL primer, 45 ng of the first-strand cDNA, and water. Primers were predesigned TaqMan Gene expression assays (S5 Table). The qPCR results were analyzed using LightCycler 96 analysis software 1.1 (Roche). The comparative threshold cycle method for relative quantification was used with *ACTIN8* (At1g49240, TaqMan At02270958).

For in—gel expression screening, total RNA was extracted using DNA-free RNA isolation protocol [74]. Isolated RNA was treated with DNase I (Invitrogen, Carlsbad, CA, USA) (Life Technologies) and precipitated by ammonium acetate (7.5 M) and ethanol. First-strand cDNA synthesis was performed using Superscript III reverse transcriptase (Invitrogen, Carlsbad, CA, USA) in a 10 μL reaction mixture containing gene specific primers. PCR amplification was done using DreamTaq DNA Polymerase (5 U/μl) (Thermo Fisher Scientific, Carlsbad, CA, USA). Primers for RT-PCR amplifications are listed in S5 Table.

### RNA-seq

Rosette leaves from soil-grown plants (4 weeks old) and seedlings grown on ½ MS with 1% sucrose (with fully expanded cotyledons) from WT Col-0 and the *psy2l* were used for RNA-seq analyses. Harvested tissue was frozen in liquid nitrogen. Total RNA was extracted using RNAeasy Plant Mini Kit and treated with on-column DNaseI digestion. Library preparation and RNA sequencing were performed by GATC Biotech (Konstanz, Germany). Expression analysis was performed by GATC Biotech using Bowtie transcriptome alignments, TopHat and Cufflink. Expression values are listed as means of three mutant samples compared with three WT samples. FPKM (fragment per kilobase per million mapped reads), and fold change with p-values are listed for significant different expression values in the mutant and WT (p<0.05) (S6 and S7 Tables).

Three replicates of each tissue type were sequenced. AgriGo (Go Analysis Toolkit and Database for Agricultural Community) [26] singular enrichment analysis (SEA) were used to facilitate identification of gene groups with altered expression in the *psy2l* mutant relative to WT. Default setting was used (Statistical test method Fisher, significance level p < 0.05). Significance values for specific gene groups are given in Table 1. A Perl script was written, which allowed to extract selected genes from the AgriGO files.

## Supporting information

S1 Fig Identification of *psy2l* knockout SALK and SAIL T-DNA lines. **A-D**, The far left pots have WT only, other pots contain mutants heterozygous or homozygous for the T-DNA insert. Arrows indicate plants homozygous for the inserts. The dwarf phenotype was observed for the SALK (A) and three SAIL lines (B-D). Plants had been grown for two months.
(PDF)

S2 Fig *Psy2l* mutants grown for 5 months. The three SAIL lines had a bushy appearance and were very small, like the SALK_048064 line. Plants continued to grow long after the WT plants had wilted, but produced only very few seeds. From left to right: SAIL_256_C08, SAIL_ 33_H01, SAIL_1275_F05. Pots are 9 cm wide.
(PDF)

S3 Fig Pollen appearance and germination. **A**, **B**, Analysis of the morphology of mature pollen grains of WT and *psy2l* mutant plants (SALK_048064). In WT most of the pollen have prolate (ovoid) morphology with tricolpate aperture (three furrows), while in *psy2l* most of the pollen did not develop mature pollen morphology. **C**, **D**, Germination of WT and *psy2l* pollen grains in the optimum solid medium. Generally, anthers of *psy2l* mutant produced less pollen grain and they were less dehiscent compared with WT. In conclusion, much less pollen germinated from *psy2l* in comparison with WT. Scale bars = 1 mm.
(PDF)

S4 Fig *Psy2l* roots have smaller meristem zone and some dead cells. Propidium iodide-stained root tips of WT and *psy2l* mutant (SALK_048064) seedlings grown on MS medium for 10 days. The arrows indicate the boundary of the meristematic zone from the quiescent center to the first elongated cell row of the transition zone, this zone was clearly smaller in *psy2l*. The cells with complete internalization of propidium iodide (red) indicate dead cells, and were visible in *psy2l*. Scale bars = 100 μm.
(PDF)

S5 Fig Representative images for the *PSY2L* knock-down amiRNA stable transgenic lines. **A**, Five and **B**, eight weeks old plants showed differences between control plants (EV/Col-0) and the two amiRNA transgenic lines that were designed to knock down *PSY2L*. Although not dwarf as the *psy2l* (T-DNA knockout mutant), the amiRNA plants showed clearly delayed flowering, distinct leaf shape, and shorter roots. **C**, Mean primary root length of six days old seedlings which were allowed to grow vertically. Mean values and standard errors are depicted, n = 30. Compared with the EV/Col-0 control, roots were significantly shorter in *psy2l* (SALK_048064) and both amiRNA lines, *p<0.001.
(PDF)

S6 Fig Semi-quantitative in-gel expression analysis of the *PP4R2L* gene in the SALK_093041 line and amiRNA lines using three different primer pairs. **A**, Relative *PP4R2L* transcript levels in the SALK_093041 line showing knockdown of the full length *PP4R2*. (**1**) primer pair spans full CDS, one plant; (**2**) primer pair targets downstream of T-DNA insert, two plants; (**3**) primer pair targets upstream of T-DNA insert, two plants. **B**, Relative *PP4R2L* transcript levels in amiRNA lines. Seeds from plants transformed by amiRNA encoding gene in binary vector were allowed to grow on the selectable marker, and positive plants were transferred to soil. Expression analysis was performed on eight transformed plants (P1-P8) using primers for full-length CDS and 200 ng of RNA. WT Col-0 and empty vector (EV)/Col-0 showed similar expression. P1 and P3 showed complete knockout of *PP4R2L*. Gel bands were semi-quantitatively measured using ImageJ (https://imagej.nih.gov/ij/index.html), and the identified peak areas were divided by Col-0 in order to show relative expression in percent.
(PDF)

S7 Fig *PSY2L* knockout and knockdown mutants show severe growth retardation upon cisplatin treatment. After growing three days on ½ MS medium with 1% sucrose, seedlings were transferred to new media for another 12 d for treatment with 0, 2, 4, or 6 mg L^-1^ cisplatin (See main text Fig 4). Percentage of seedlings showing strong growth retardation was visually observed. The experiments were repeated 3 times, and totally there were 30–40 seedlings for each plant type and concentration of cisplatin. SE is given. EV/Col-0 and *pp4r2l-ami* seedlings at 2 mg L^-1^ cisplatin were not different from zero cisplatin control (ns, not significant), but *psy2l* (SALK) and *psy-ami* seedlings showed significant growth retardation at 2 mg L^-1^ cisplatin, p<0.01. Higher cisplatin concentrations (4–6 mg L^-1^) gave growth retardation for all seedlings at p<0.01.
(PDF)

S8 Fig Subcellular targeting analysis for PP4 catalytic and regulatory subunits fused both N and C-terminally to EYFP and analyzed in Arabidopsis mesophyll protoplasts. **A**, Free N-terminus PSY2L targeted only nucleus. **B**, free C-terminus PSY2L targeted nucleus and cytosol. **C**, **D**, PP4R2L targeted mostly cytosol. **E-I**, cytosolic and weak nucleus targeting for PP4.1. **J-K**, PP4.2 targeting to cytosol, nucleus, and unknown punctate structures. Endoplasmic reticulum was labeled by OFP-ER [25]. Chloroplasts were captured by chlorophyll autofluorescence. Arrows point to the presence of nucleus (N). Scale bars = 10 μM.
(PDF)

S1 Table Genes two-fold up-regulated in *psy2l* rosette leaves (annotated). (XLSX)

S2 Table Genes two-fold down-regulated in *psy2l* rosette leaves (annotated). (XLSX)

S3 Table Genes two-fold up-regulated in *psy2l* seedlings (annotated). (XLSX)

S4 Table Genes two-fold down-regulated in *psy2l* seedlings (annotated). (XLSX)

S5 Table List of primers. (PDF)

S6 Table All genes significant differently expressed in *psy2l* and WT rosette leaves. (XLSX)

S7 Table All genes significant differently expressed in in *psy2l* and WT seedlings. (XLSX)
