# Supplementary material for: PLATINUM SENSITIVE 2 LIKE impacts growth, root morphology, seed set, and stress responses
Source: PLoS One. 2017 Jul 5;12(7):e0180478. doi: 10.1371/journal.pone.0180478 (PMC5498030; doi:10.1371/journal.pone.0180478)
Supplement: S1 Fig — A-D, The far left pots have WT only, other pots contain mutants heterozygous or homozygous for the T-DNA insert. Arrows indicate plants homozygous for the inserts. The dwarf phenotype was observed for the SALK (A) and three SAIL lines (B-D). Plants had been grown for two months. (PDF) [file pone.0180478.s001.pdf]

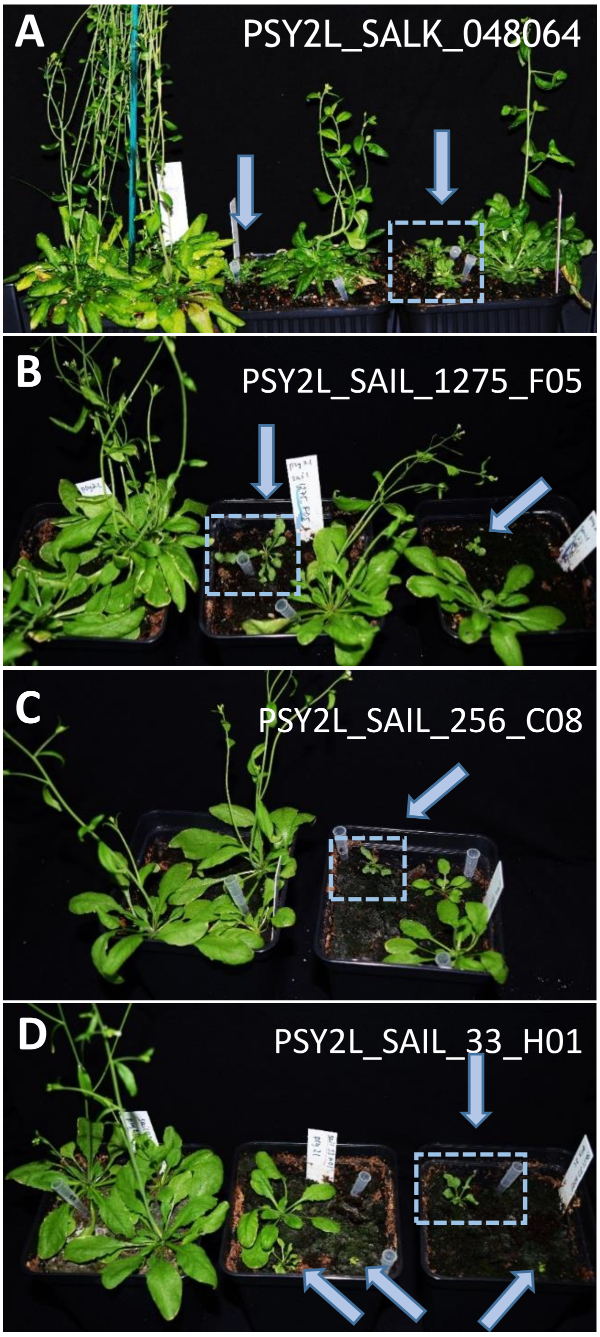

S1 Fig. Identification of *psy2l* knockout SALK and SAIL T-DNA lines.
A-D, The far left pots have WT only, other pots contain mutants heterozygous or homozygous for the T-DNA insert. Arrows indicate plants homozygous for the inserts. The dwarf phenotype was observed for the SALK (A) and three SAIL lines (B-D). Plants had been grown for two months.
