# Supplementary material for: PLATINUM SENSITIVE 2 LIKE impacts growth, root morphology, seed set, and stress responses
Source: PLoS One. 2017 Jul 5;12(7):e0180478. doi: 10.1371/journal.pone.0180478 (PMC5498030; doi:10.1371/journal.pone.0180478)
Supplement: S2 Fig — The three SAIL lines had a bushy appearance and were very small, like the SALK_048064 line. Plants continued to grow long after the WT plants had wilted, but produced only very few seeds. From left to right: SAIL_256_C08, SAIL_ 33_H01, SAIL_1275_F05. Pots are 9 cm wide. (PDF) [file pone.0180478.s002.pdf]

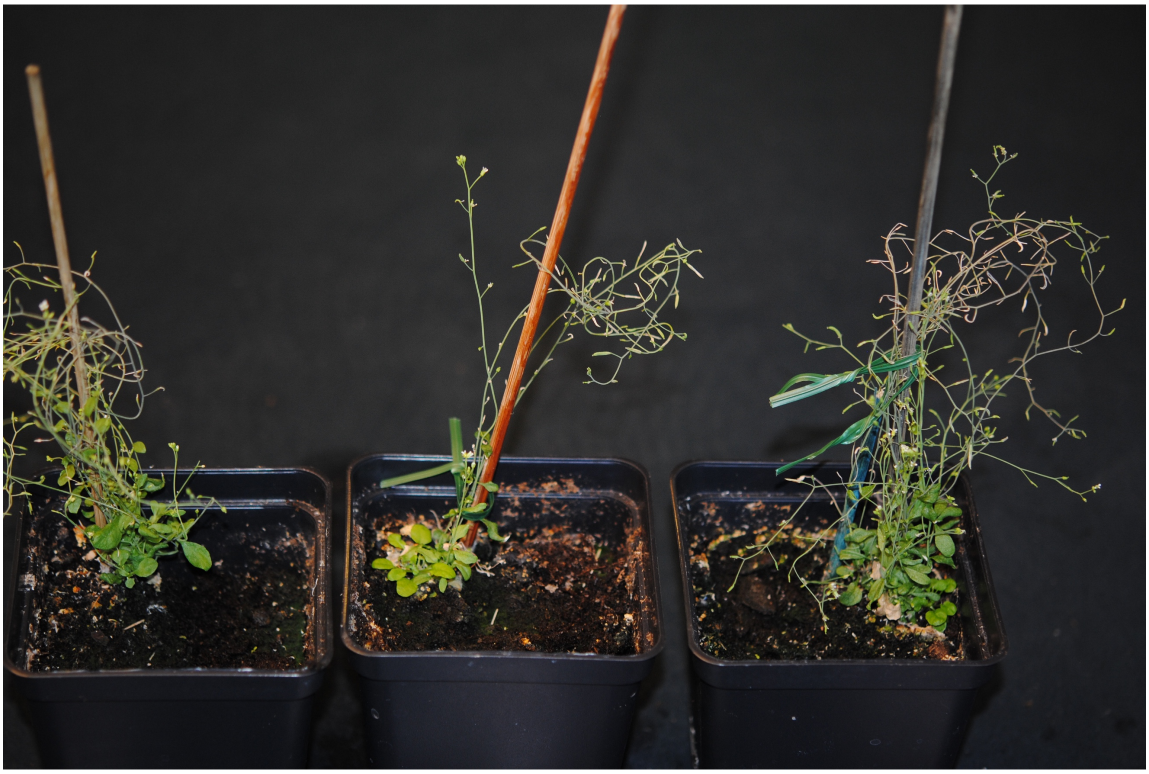

S2 Fig. ***Psy2l*** **mutants grown for 5 months.**
The three SAIL lines had a bushy appearance and were very small, like the SALK_048064 line. Plants continued to grow long after the WT plants had wilted, but produced only very few seeds. From left to right: SAIL_256_C08, SAIL_ 33_H01, SAIL_1275_F05. Pots are 9 cm wide.
