# Supplementary material for: PLATINUM SENSITIVE 2 LIKE impacts growth, root morphology, seed set, and stress responses
Source: PLoS One. 2017 Jul 5;12(7):e0180478. doi: 10.1371/journal.pone.0180478 (PMC5498030; doi:10.1371/journal.pone.0180478)
Supplement: S3 Fig — A, B, Analysis of the morphology of mature pollen grains of WT and psy2l mutant plants (SALK_048064). In WT most of the pollen have prolate (ovoid) morphology with tricolpate aperture (three furrows), while in psy2l most of the pollen did not develop mature pollen morphology. C, D, Germination of WT and psy2l pollen grains in the optimum solid medium. Generally, anthers of psy2l mutant produced less pollen grain and they were less dehiscent compared with WT. In conclusion, much less pollen germinated from psy2l in comparison with WT. Scale bars = 1 mm. (PDF) [file pone.0180478.s003.pdf]

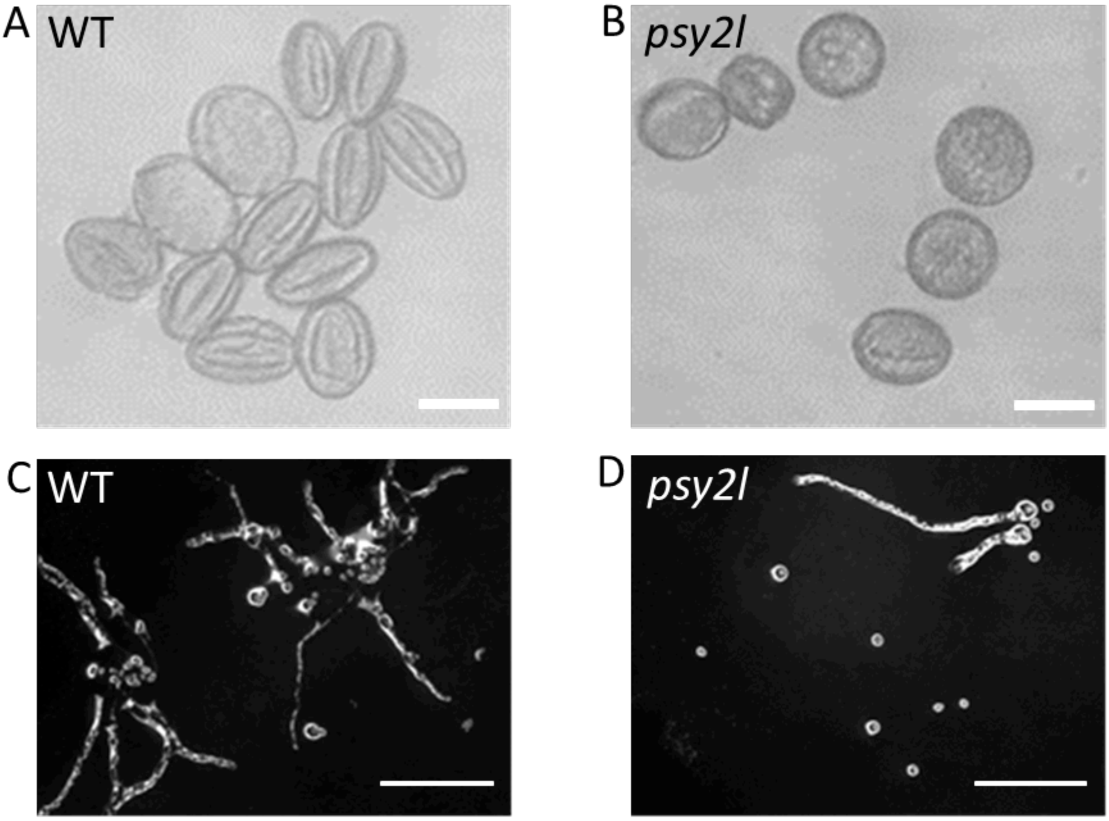

S3 Fig. Pollen appearance and germination.
A, **B**, Analysis of the morphology of mature pollen grains of WT and *psy2l* mutant plants (SALK_048064). In WT most of the pollen have prolate (ovoid) morphology with tricolpate aperture (three furrows), while in *psy2l* most of the pollen did not develop mature pollen morphology.
C, **D**, Germination of WT and *psy2l* pollen grains in the optimum solid medium. Generally, anthers of *psy2l* mutant produced less pollen grain and they were less dehiscent compared with WT. In conclusion, much less pollen germinated from *psy2l* in comparison with WT. Scale bars = 1 mm.

Pollen was germinated on agar medium containing 18% sucrose, 0.01% boric acid, 1 mM $MgSO_4$, 1 mM $CaCl_2$, 1 mM $Ca(NO_3)_2$, and 0.5% agar, pH 7. Open flowers were dehydrated at room temperature for at least 2 h. Pollen grains were shed to solid agar medium by touching the flowers against agar medium. Pollen was germinated at room temperature overnight, examined under a light microscope, and photographed with Leica M80 stereomicroscope (Method after: Li H, Lin Y, Heath RM, Zhu MX, Yang Z. Control of pollen tube tip growth by a Rop GTPase-dependent pathway that leads to tip-localized calcium influx. Plant Cell. 1999;11(9):1731-42).
