# Supplementary material for: PLATINUM SENSITIVE 2 LIKE impacts growth, root morphology, seed set, and stress responses
Source: PLoS One. 2017 Jul 5;12(7):e0180478. doi: 10.1371/journal.pone.0180478 (PMC5498030; doi:10.1371/journal.pone.0180478)
Supplement: S4 Fig — Propidium iodide-stained root tips of WT and psy2l mutant (SALK_048064) seedlings grown on MS medium for 10 days. The arrows indicate the boundary of the meristematic zone from the quiescent center to the first elongated cell row of the transition zone, this zone was clearly smaller in psy2l. The cells with complete internalization of propidium iodide (red) indicate dead cells, and were visible in psy2l. Scale bars = 100 μm. (PDF) [file pone.0180478.s004.pdf]

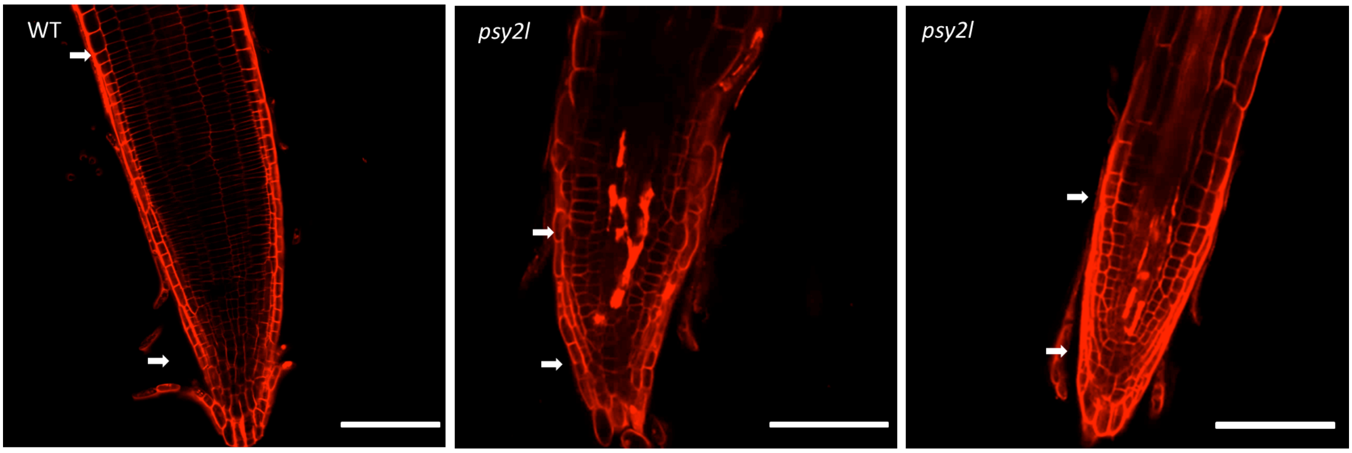

S4 Fig. *Psy2l* roots have smaller meristem zone and some dead cells.
Propidium iodide-stained root tips of WT and *psy2l* mutant (SALK_048064) seedlings grown on MS medium for 10 days. The arrows indicate the boundary of the meristematic zone from the quiescent center to the first elongated cell row of the transition zone, this zone was clearly smaller in *psy2l.* The cells with complete internalization of propidium iodide (red) indicate dead cells, and were visible in *psy2l.* Scale bars = 100 µm.

For root tip meristemic zone and dead cells detection, roots of 10 days old seedlings grown on ½ MS media were stained with 10 µg mL^{-1} propidium iodide (Sigma Aldrich, USA). Excitation and emission wavelengths were set as 535 nm and 617 nm, respectively, for fluorescence microscopy (Method after: Zhang HL, Gu ZY, Wu Q, Yang LF, Liu CF, Ma H, et al. Arabidopsis PARG1 is the key factor promoting cell survival among the enzymes regulating post-translational poly(ADP-ribosyl)ation. Sci Rep. 2015;5. doi: ARTN 15892 10.1038/srep15892).
