# Supplementary material for: PLATINUM SENSITIVE 2 LIKE impacts growth, root morphology, seed set, and stress responses
Source: PLoS One. 2017 Jul 5;12(7):e0180478. doi: 10.1371/journal.pone.0180478 (PMC5498030; doi:10.1371/journal.pone.0180478)
Supplement: S5 Fig — A, Five and B, eight weeks old plants showed differences between control plants (EV/Col-0) and the two amiRNA transgenic lines that were designed to knock down PSY2L. Although not dwarf as the psy2l (T-DNA knockout mutant), the amiRNA plants showed clearly delayed flowering, distinct leaf shape, and shorter roots. C, Mean primary root length of six days old seedlings which were allowed to grow vertically. Mean values and standard errors are depicted, n = 30. Compared with the EV/Col-0 control, roots were significantly shorter in psy2l (SALK_048064) and both amiRNA lines, *p<0.001. (PDF) [file pone.0180478.s005.pdf]

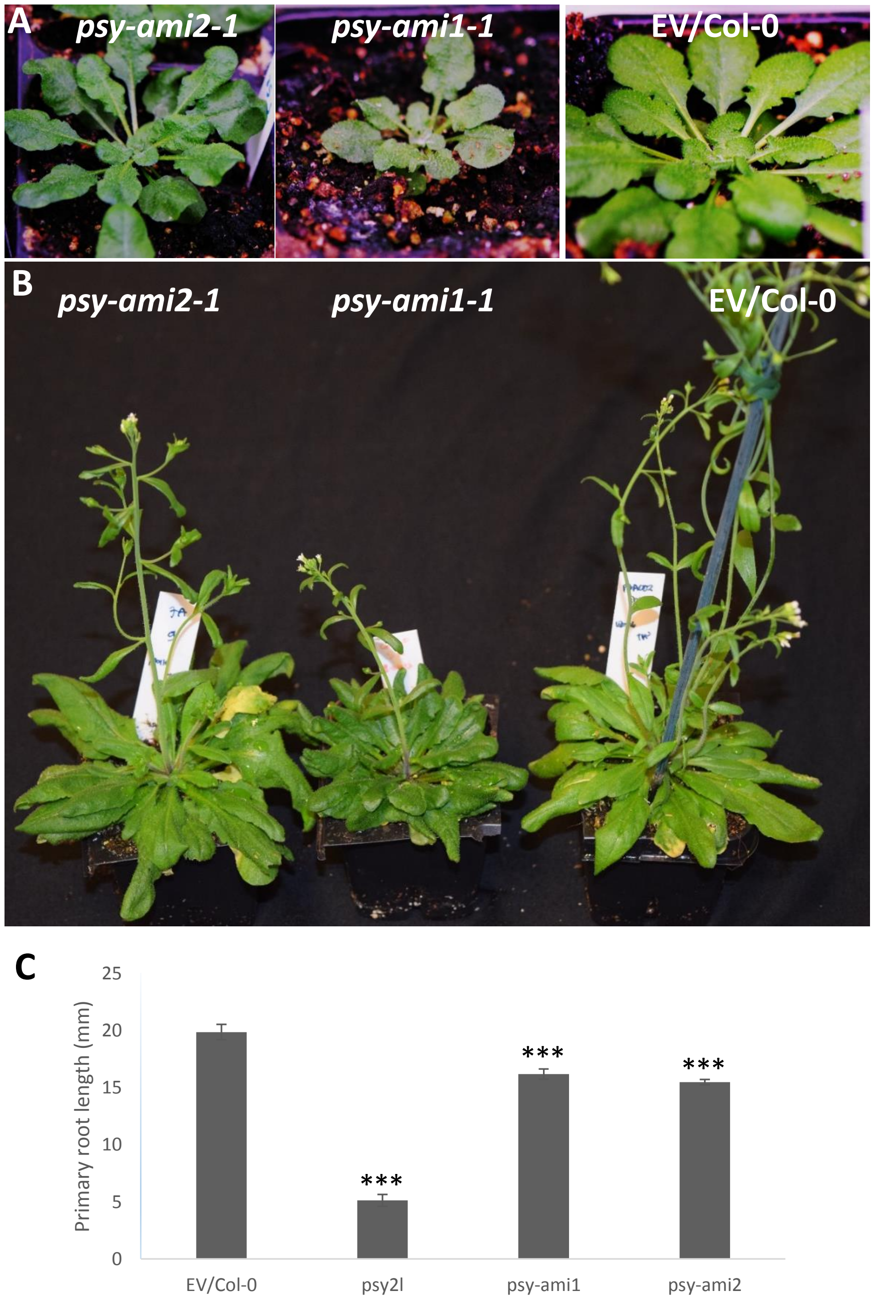

S5 Fig. Representative images for the *PSY2L* knock-down amiRNA stable transgenic lines.
A, Five and **B**, eight weeks old plants showed differences between control plants (EV/Col-0) and the two amiRNA transgenic lines that were designed to knock down *PSY2L*. Although not dwarf as the *psy2l* (T-DNA knockout mutant), the amiRNA plants showed clearly delayed flowering, distinct leaf shape, and shorter roots. **C**, Mean primary root length of six days old seedlings which were allowed to grow vertically. Mean values and standard errors are depicted, n=30. Compared with the EV/Col-0 control, roots were significantly shorter in *psy2l* (SALK_048064) and both amiRNA lines, *p<0.001.
