# Supplementary material for: PLATINUM SENSITIVE 2 LIKE impacts growth, root morphology, seed set, and stress responses
Source: PLoS One. 2017 Jul 5;12(7):e0180478. doi: 10.1371/journal.pone.0180478 (PMC5498030; doi:10.1371/journal.pone.0180478)
Supplement: S6 Fig — A, Relative PP4R2L transcript levels in the SALK_093041 line showing knockdown of the full length PP4R2. (1) primer pair spans full CDS, one plant; (2) primer pair targets downstream of T-DNA insert, two plants; (3) primer pair targets upstream of T-DNA insert, two plants. B, Relative PP4R2L transcript levels in amiRNA lines. Seeds from plants transformed by amiRNA encoding gene in binary vector were allowed to grow on the selectable marker, and positive plants were transferred to soil. Expression analysis was performed on eight transformed plants (P1-P8) using primers for full-length CDS and 200 ng of RNA. WT Col-0 and empty vector (EV)/Col-0 showed similar expression. P1 and P3 showed complete knockout of PP4R2L. Gel bands were semi-quantitatively measured using ImageJ (https://imagej.nih.gov/ij/index.html), and the identified peak areas were divided by Col-0 in order to show relative expression in percent. (PDF) [file pone.0180478.s006.pdf]

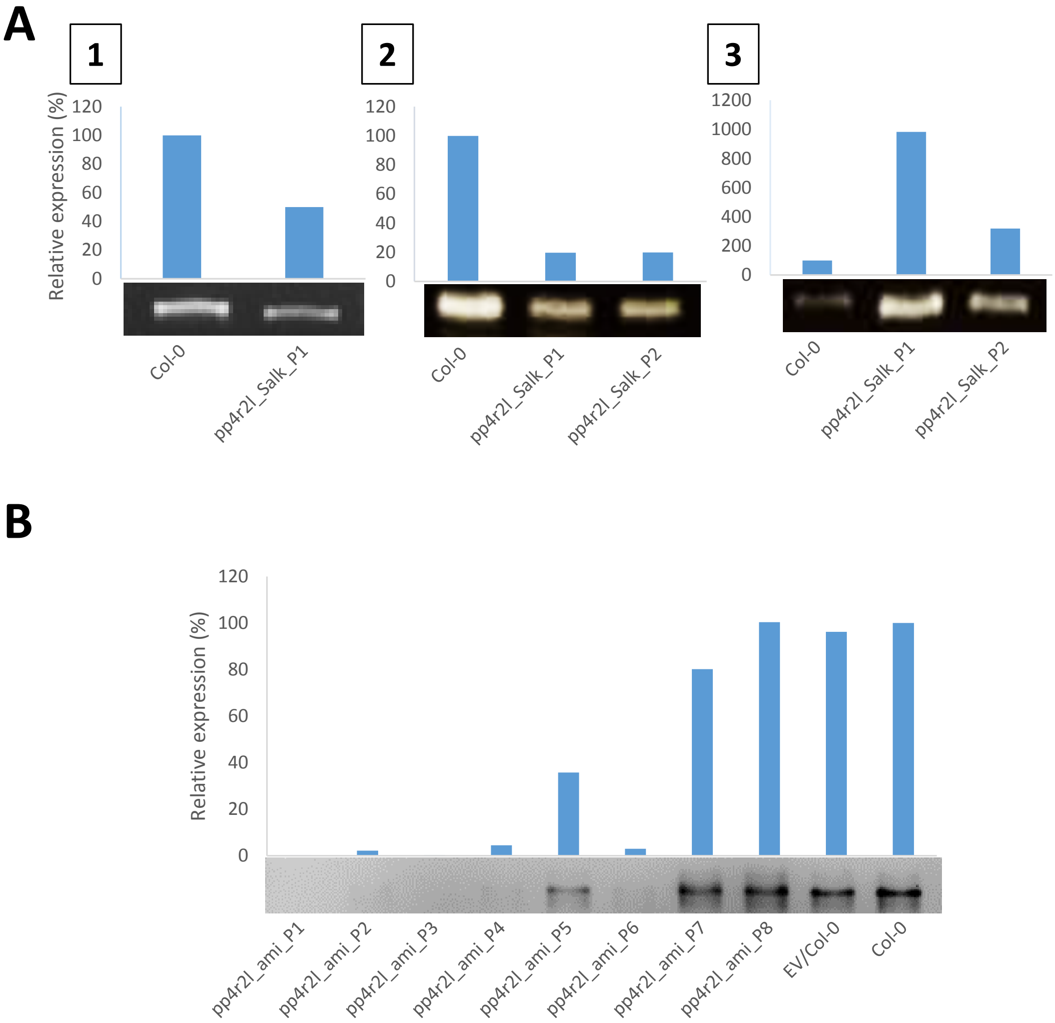

S6 Fig. Semi-quantitative in-gel expression analysis of the *PP4R2L* gene in the SALK_093041 line and amiRNA lines using three different primer pairs. A, Relative *PP4R2L* transcript levels in the SALK_093041 line showing knockdown of the full length *PP4R2*. (**1**) primer pair spans full CDS, one plant; (**2**) primer pair targets downstream of T-DNA insert, two plants; (**3**) primer pair targets upstream of T-DNA insert, two plants. **B,** Relative *PP4R2L* transcript levels in amiRNA lines**.** Seeds from plants transformed by amiRNA encoding gene in binary vector were allowed to grow on the selectable marker, and positive plants were transferred to soil. Expression analysis was performed on eight transformed plants (P1-P8) using primers for full-length CDS and 200 ng of RNA. WT Col-0 and empty vector (EV)/Col-0 showed similar expression. P1 and P3 showed complete knockout of *PP4R2L*. Gel bands were semi-quantitatively measured using ImageJ (https://imagej.nih.gov/ij/index.html), and the identified peak areas were divided by Col-0 in order to show relative expression in percent.
