# Supplementary material for: PLATINUM SENSITIVE 2 LIKE impacts growth, root morphology, seed set, and stress responses
Source: PLoS One. 2017 Jul 5;12(7):e0180478. doi: 10.1371/journal.pone.0180478 (PMC5498030; doi:10.1371/journal.pone.0180478)
Supplement: S7 Fig — After growing three days on ½ MS medium with 1% sucrose, seedlings were transferred to new media for another 12 d for treatment with 0, 2, 4, or 6 mg L-1 cisplatin (See main text Fig 4). Percentage of seedlings showing strong growth retardation was visually observed. The experiments were repeated 3 times, and totally there were 30–40 seedlings for each plant type and concentration of cisplatin. SE is given. EV/Col-0 and pp4r2l-ami seedlings at 2 mg L-1 cisplatin were not different from zero cisplatin control (ns, not significant), but psy2l (SALK) and psy-ami seedlings showed significant growth retardation at 2 mg L-1 cisplatin, p<0.01. Higher cisplatin concentrations (4–6 mg L-1) gave growth retardation for all seedlings at p<0.01. (PDF) [file pone.0180478.s007.pdf]

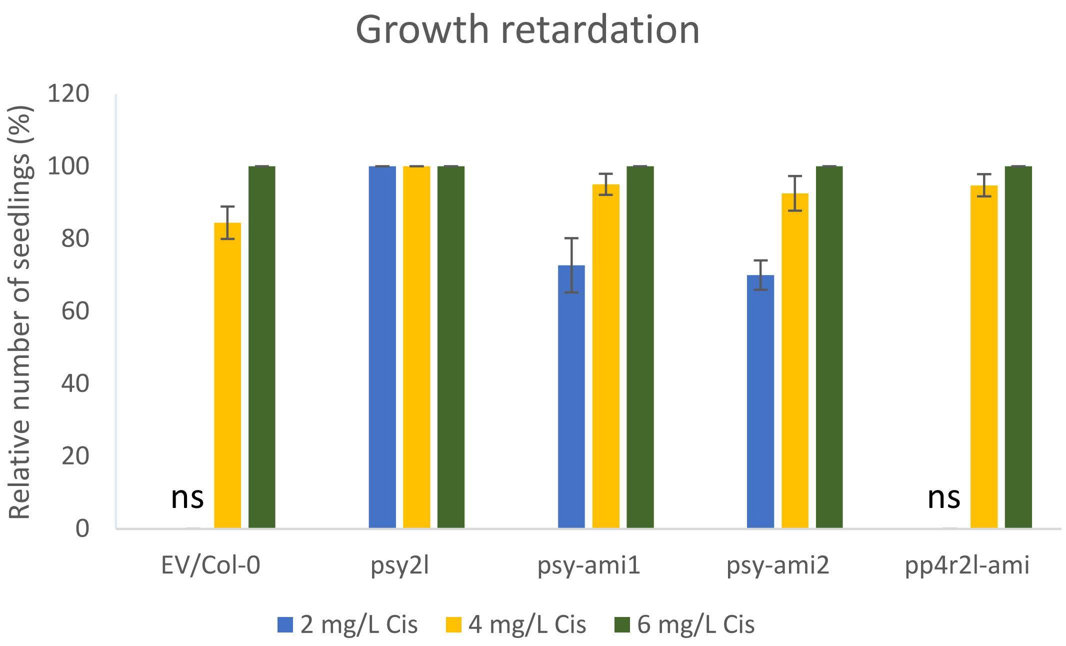

S7 Fig. *PSY2L* knockout and knockdown mutants show severe growth retardation upon cisplatin treatment. After growing three days on ½ MS medium with 1% sucrose, seedlings were transferred to new media for another 12 d for treatment with 0, 2, 4, or 6 mg L^{-1} cisplatin (See main text Fig 4). Percentage of seedlings showing strong growth retardation was visually observed. The experiments were repeated 3 times, and totally there were 30-40 seedlings for each plant type and concentration of cisplatin. SE is given. EV/Col-0 and *pp4r2l-ami* seedlings at 2 mg L^{-1} cisplatin were not different from zero cisplatin control (ns, not significant), but *psy2l* (SALK) and *psy-ami* seedlings showed significant growth retardation at 2 mg L^{-1} cisplatin, $p<0.01$. Higher cisplatin concentrations (4-6 mg L^{-1}) gave growth retardation for all seedlings at $p<0.01$.
