# Supplementary material for: PLATINUM SENSITIVE 2 LIKE impacts growth, root morphology, seed set, and stress responses
Source: PLoS One. 2017 Jul 5;12(7):e0180478. doi: 10.1371/journal.pone.0180478 (PMC5498030; doi:10.1371/journal.pone.0180478)
Supplement: S8 Fig — A, Free N-terminus PSY2L targeted only nucleus. B, free C-terminus PSY2L targeted nucleus and cytosol. C, D, PP4R2L targeted mostly cytosol. E-I, cytosolic and weak nucleus targeting for PP4.1. J-K, PP4.2 targeting to cytosol, nucleus, and unknown punctate structures. Endoplasmic reticulum was labeled by OFP-ER [25]. Chloroplasts were captured by chlorophyll autofluorescence. Arrows point to the presence of nucleus (N). Scale bars = 10 μM. (PDF) [file pone.0180478.s008.pdf]

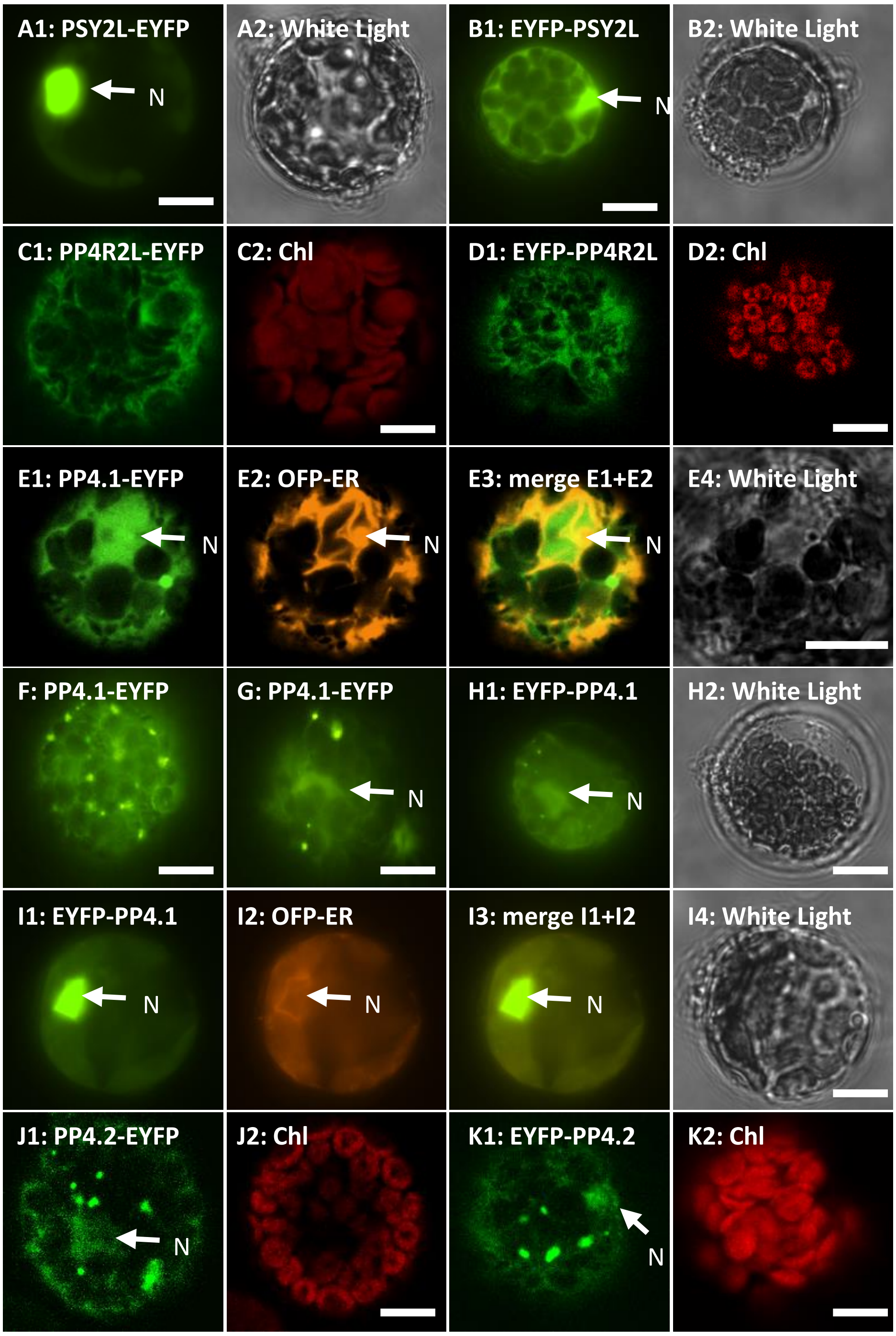


**S8 Fig. Subcellular targeting analysis for PP4 catalytic and regulatory subunits fused both N and C-terminally to EYFP and analyzed in Arabidopsis mesophyll protoplasts.**
**A**, Free N-terminus PSY2L targeted only nucleus. **B**, free C-terminus PSY2L targeted nucleus and cytosol. **C**, **D**, PP4R2L targeted mostly cytosol. **E-I**, cytosolic and weak nucleus targeting for PP4.1. **J-K**, PP4.2 targeting to cytosol, nucleus, and unknown punctate structures. Endoplasmic reticulum was labeled by OFP-ER [25]. Chloroplasts were captured by chlorophyll autofluorescence. Arrows point to the presence of nucleus (N). Scale bars = 10 μM
