# Supplementary material for: PLATINUM SENSITIVE 2 LIKE impacts growth, root morphology, seed set, and stress responses
Source: PLoS One. 2017 Jul 5;12(7):e0180478. doi: 10.1371/journal.pone.0180478 (PMC5498030; doi:10.1371/journal.pone.0180478)
Supplement: S5 Table — (PDF) [file pone.0180478.s013.pdf]

**Subcellular localization primers**						
**Gene**	**AGI**	**Name**	**Primer**	**Fusion protein**	**R.S.**	**Template**
PP4-1	At4g26720	AK72F	ATGCGGCCGCTATGTCAGACCTAGATCGGCAAA	EYFP-PP4-1	NotI	PP4-1/Ws Jose Serrano/CNB
		AK73R	ATCCGCGGTTATAGGAAGTAATCAGGGG	EYFP-PP4-1	SacII	
		AK74F	ATGAGCTCTCATGTCAGACCTAGATCGGCA	PP4-1-EYFP	SacI	
		AK75R	ATGCGGCCGCTAGGAAGTAATCAGGGGCCG	PP4-1-EYFP	NotI	
PP4-2	At5g55260	AK76F	ATGCGGCCGCTATGTCAGACCTAGACAAGCAA	EYFP-PP4-2	NotI	U83558
		as AK73R	ATCCGCGGTCACAGGAAATAATCAGGTG	EYFP-PP4-2	SacII	
		AK77F	ATCCATGGGTATGTCAGACCTAGACAAGCA	PP4-2-EYFP	NcoI	
		as AK75R	ATGCGGCCGCCAGGAAATAATCAGGTGCAG	PP4-2-EYFP	NotI	
PP4-1		AK74F_b2014	ATGAGCTCTCATGTCAGACCTAGATCGGCAAATAG	PP4-1-EYFP	SacI	
		AK75R_b2014	ATGCGGCCGCTAGGAAGTAATCAGGGGCCGGCTT	EYFP-PP4-1 or 2	NotI	
PP4-2		AK77F_b2014	ATCCATGGGTATGTCAGACCTAGACAAGCAAATAGAG	EYFP-PP4-2	NcoI	
PP4R2L	At5g17070	EYFP_PP4R2 (At5g17070.1)F	AAAGCGGCCGCTATGGAGAATCCGTCATCATCG	EYFP_PP4R2L	NotI	U24915
		EYFP_PP4R2 (At5g17070.1)R	ATTCCGCGGCTAGGCACACGTTGTAGGCAA	EYFP_PP4R2L	SacII	
		PP4R2_EYFP (At5g17070.1)F	ATCCATGGGTATGGAGAATCCGTCATCATCG	PP4R2L_EYFP	NcoI	
		PP4R2_EYFP(At5g17070.1)R	AAGCGGCCGCGGCACACGTTGTAGGCAACCG	PP4R2L_EYFP	NotI	
PSY2L	At3g06670	EYFP_PSY2L (At3g06670.1)F	AAAGCGGCCGCTATGGGCGCTCCGGAAAAGTCT	EYFP_PSY2L	NotI	U21916
		EYFP_PSY2L (At3g06670.1)R	ATTGCGGCCGCTCAGGATCCATTTACAGCCAT	EYFP_PSY2L	NotI	
		PSY2L_EYFP(At3g06670.1)F	ATCCATGGGTATGGGCGCTCCGGAAAAGTCT	PSY2L_EYFP	NcoI	
		PSY2L_EYFP (At3g06670.1)R	ATTGCGGCCGCGGATCCATTTACAGCCAT	PSY2L_EYFP	NotI	
**T_DNA genotyping primers**						
		PP4-1-(SALK_070977)-LP	TTGTATCCAGCCCGTTATTTG			
		PP4-1-(SALK_070977)-RP	AGAATTCCGTGTGTTTGATGC			
		PP4-1.(GK_651B07)-LP	ACAAAATCAAAGACACGGTCG			
		PP4-1.(GK-651B07)-RP	TAAAGACACAAAATCTCCGCG			
		PP4-2-(SAIL_569_H09)-LP	TGGAGAGAGACCTCCATGAAC			
		PP4-2-(SAIL_569_H09)-RP	ATAGAGCAGCTTAAACGCTGC			
		PP4-2-(SALK_049725C)LP	TTCATGGAGGTCTCTCTCCAG			
		PP4-2-(SALK_049725C)RP	AACGATGACCTTGCAATTTTG			
		PSY2L_SALK_048064_LP	ATGTTTCGCCTGTTCAATCAC			
		PSY2L_SALK_048064_RP	TGTTGAATTGAGATGGAAGGG			
		PSY2L_SALK_125872_LP	AAGCCTCTGAGGATGAGGAAG			
		PSY2L_SALK_125872_RP	AAATGAATATGGCTTTTGGGG			
		PSY2L_SAIL_1275_F05_LP	AGGCGCATCTGATCTGTAATG			
		PSY2L_SAIL_1275_F05_RP	CTTTCAAGAGACTGCAGGGTG			
		PSY2L_SAIL_33_H01_LP	TCTCTTCTCTCTTCTTTCGCG			
		PSY2L_SAIL_33_H01_RP	AATATCCTCAGGGTTGATGGG			
		PSY2L_SAIL_256_C08_LP	TGTACCGTTTTTGAATCTGTGC			
		PSY2L_SAIL_256_C08_RP	GTGCTCAAGCAGATCCAAGAC			
		PP4R2L_SALK_093041_LP	CAACATATTTGGCATTTTGGC			
		PP4R2L_SALK_093041_RP	TGTTCAACAGATCCTTTTGGC			
		LB1 for SAIL C/418-451	GCCTTTTCAGAAATGGATAAATAGCCTTGCTTCC			
		LBa1 for SALK/pBIN-pROK2	TGGTTCACGTAGTGGGCCATCG			
		LBb1.3 for SALK/pBIN-pROK2	ATTTTGCCGATTTCGGAAC			
		8474_ GABI-Kat LB-FSTs	ATAATAACGCTGCGGACATCTACATTTT			

**On gel RT-PCR primers**						
PP4-1		AK108-F_PP4-1_RT	TTTGACTACATGAGTCTTTCAGCTGTTG			
		AK109-R_PP4-1_RT	TTGATGGGCACGGGCTATGTAGTCT			
PP4-2		AK110-F_PP4-2_RT	TTCTAGCTCTCAAGGTTAGATATCCAGAC			
		AK111-R_PP4-2_RT	TAGCTGATGAGCTCGACATATGTAATCA			
PP4R2L		RT_PP4R2L_Front1_ LP	GAGATTTCCTCCGTCGTTCA			
		RT_PP4R2L _Front1_RP	ATCCGTCATTTTTGCCTCAG			
		RT_PP4R2L_Back2_LP	GCAATGACTGTTGACATGGA			
		RT_PP4R2L _Back2_RP	TAGGCAACCGTGAATCTCCT			
		RT_PP4R2L_Back3_LP	TGGAAACAATCGATGAACCA			
		RT_PP4R2L_Back3_RP	AAACCAGAAATTCCACAAAAACA			
	Full CDS	EYFP_PP4R2 (At5g17070.1)F	AAAGCGGCCGCTATGGAGAATCCGTCATCATCG			
	Full CDS	EYFP_PP4R2 (At5g17070.1)R	ATTCCGCGGCTAGGCACACGTTGTAGGCAA			
PSY2L		RT_PSY2L_LP	CTCATGGAGAGACCCAGAGC			
		RT_PSY2L_RP	CCATCAGATTCCGGAAGAAA			
**Stable amiRNA lines**						
**amiRNA**				**amiRNA seuence**		**Binary vector**
**1**	PP4-1 + PP4-2	1-I miR-s	GATAATGAGAGTTATACGGTCTATCTCTCTTTTGTATTCC	**TAATGAGAGTTATACGGTCTA**		pBA002
		2-II miR-a	GATAGACCGTATAACTCTCATTATCAAAGAGAATCAATGA			
		3-III miR*s	GATAAACCGTATAACACTCATTTTCACAGGTCGTGATATG			
		4-IV miR*a	GAAAATGAGTGTTATACGGTTTATCTACATATATATTCCT			
**2**	PP4-1 + PP4-2	5-I miR-s	GATTAAAAGACGTAACAACGCTGTCTCTCTTTTGTATTCC	**TTAAAAGACGTAACAACGCTG**		pBA002
		6-II miR-a	GACAGCGTTGTTACGTCTTTTAATCAAAGAGAATCAATGA			
		7-III miR*s	GACAACGTTGTTACGACTTTTATTCACAGGTCGTGATATG			
		8-IV miR*a	GAATAAAAGTCGTAACAACGTTGTCTACATATATATTCCT			
**1**	PSY2L	I miR-s	GATAAATAAGTTCAGTTAGGCGTTCTCTCTTTTGTATTCC	**TAAATAAGTTCAGTTAGGCGT**		pBA002
		II miR-a	GAACGCCTAACTGAACTTATTTATCAAAGAGAATCAATGA			
		III miR*s	GAACACCTAACTGAAGTTATTTTTCACAGGTCGTGATATG			
		IV miR*a	GAAAAATAACTTCAGTTAGGTGTTCTACATATATATTCCT			
**2**	PSY2L	I miR-s	GATTACTATGGCATTGTTCGCACTCTCTCTTTTGTATTCC	**TTACTATGGCATTGTTCGCAC**		pBA002
		II miR-a	GAGTGCGAACAATGCCATAGTAATCAAAGAGAATCAATGA			
		III miR*s	GAGTACGAACAATGCGATAGTATTCACAGGTCGTGATATG			
		IV miR*a	GAATACTATCGCATTGTTCGTACTCTACATATATATTCCT			
**1**	PP4R2L	I miR-s	GATATCCGATAATGGTCGTGCAGTCTCTCTTTTGTATTCC	**TATCCGATAATGGTCGTGCAG**		pBA002
		II miR-a	GACTGCACGACCATTATCGGATATCAAAGAGAATCAATGA			
		III miR*s	GACTACACGACCATTTTCGGATTTCACAGGTCGTGATATG			
		IV miR*a	GAAATCCGAAAATGGTCGTGTAGTCTACATATATATTCCT			
**Miscellanous primers**						
		C.Term.EYFPf	ACTACCTGAGCTACCAGTCC			
		AK92r/downstream EYFP	CCTTATCTGGGAACTACTCAC			
		AK93f/upstream EYFP	GCATTCTACTTCTATTGCAGC			
		AK94r/middle EYFP	AACTTCAGGGTCAGCTTGCCGT			
		NOS	GATAATCATCGCAAGACCGGCAACAGGA			
		A/amiRNA specific	CTGCAAGGCGATTAAGTTGGGTAAC			
		B/amiRNA specific	GCGGATAACAATTTCACACAGGAAACAG			
**TaqMan® Gene Expression Assay**						
Gene	AGI	Assay ID	Catalog #			

PSY2L	AT3G06670	At02236429_g1	4351372			
PSY2L	AT3G06670	At02236437_g1	4351372			
